# Influence of Carbonization and Steam Activation Temperatures on the Physicochemical and Sorption Properties of Activated Carbons Derived from Agricultural and Agri-Food Biowaste

**DOI:** 10.3390/ma19214667

**Published:** 2026-11-02

**Authors:** Ewa Szatyłowicz, Renata Weygand, Magdalena Łępicka

**Affiliations:** 1 Department of Technology in Environmental Engineering, Faculty of Civil Engineering and Environmental Sciences, Bialystok University of Technology, St. Wiejska 45A, 15-351 Bialystok, Poland; 2 Grand Activated Sp. z o.o., St. Białostocka 1, 17-200 Hajnówka, Poland; weygandr@grand-activated.pl; 3 Department of Materials and Production Engineering, Faculty of Mechanical Engineering, Bialystok University of Technology, ul. Wiejska 45 C, 15-351 Bialystok, Poland; m.lepicka@pb.edu.pl

**Keywords:** activated carbon, agricultural and agri-food waste, carbonization, steam activation, adsorption properties

## Abstract

This study investigated the influence of combined carbonization and steam activation temperatures on the physicochemical and adsorption-related properties of activated carbons derived from five agricultural and agri-food biowastes: sugar beet pulp, sunflower husk pellets, apple pomace, straw, and corn cobs. The precursors were carbonized at 400, 500, or 600 °C and subsequently activated with steam at 600, 700, or 850 °C, respectively. Apple pomace, straw, and corn cobs were pelletized using sugar beet molasses as a binder. The resulting materials were characterized in terms of process yield, iodine number, BET specific surface area, ash content, and other physicochemical parameters. Selected activated carbons were additionally examined using scanning electron microscopy (SEM) and energy-dispersive X-ray spectroscopy (EDS). Increasing thermal treatment severity generally improved the adsorption-related properties, although the response was strongly precursor-dependent. Sugar beet pulp exhibited the highest BET specific surface area (459 m^2^/g) and iodine number (477 mg/g), while corn cob-derived activated carbon reached 449 m^2^/g and 467 mg/g, respectively, after the 600/850 °C treatment sequence. SEM observations revealed limited morphological differences between sugar beet pulp samples treated at 500/700 and 600/850 °C, whereas corn cob-derived carbons exhibited more pronounced surface fragmentation and visible cavities at higher treatment severity. EDS analysis revealed distinct inorganic elemental signatures, particularly Ca in sugar beet pulp-derived carbon and K in corn cob-derived carbon. Improved adsorption-related properties were accompanied by reduced material yields, while straw exhibited limited thermal stability under the most severe treatment conditions. The findings demonstrate the importance of precursor-specific optimization of thermal processing conditions and the combined evaluation of physicochemical, morphological, and material yield characteristics.

## 1. Introduction

Agricultural and agri-food wastes are a source of raw materials for the production of carbonaceous materials with properties suitable for environmental protection applications. Their conversion into sorbents is consistent with the principles of the circular economy, enabling production residues to be transformed into products with greater functional value. In addition to biological methods of biomass management, thermochemical processes play an important role, as they enable the organic fraction to be converted into solid, liquid and gaseous products. The selection of appropriate process conditions makes it possible to direct the conversion towards the production of carbonaceous materials intended for further activation and adsorption applications [1,2]. The suitability of waste biomass for activated carbon production results primarily from the presence of cellulose, hemicelluloses and lignin, whose thermal decomposition leads to the formation of a carbon skeleton. However, individual feedstocks differ in the proportions of these components, mineral matter content and structural characteristics, which affect carbonisation yield and the susceptibility of the resulting material to activation. Therefore, the availability of waste material and its low acquisition cost are not sufficient criteria for selecting a precursor. It is also necessary to assess the possibility of obtaining appropriate porosity and the desired physicochemical properties of the final sorbent [3,4].

Biomass-derived activated carbons have attracted considerable attention as potential adsorbents for the removal of organic and inorganic contaminants from aqueous solutions, including dyes, pharmaceuticals, and selected metal ions. Their adsorption performance depends on several interrelated physicochemical characteristics, particularly the specific surface area, pore-size distribution, accessibility of adsorption sites, and surface chemical properties. The specific surface area and pore structure determine the extent to which adsorbate molecules can access the internal carbon surface, whereas surface functional groups and the chemical characteristics of the adsorbate influence the interactions occurring during adsorption. Consequently, a high BET specific surface area does not necessarily correspond to high adsorption efficiency for every pollutant, as molecular dimensions, solution conditions, and the affinity between the adsorbate and carbon surface must also be considered. Previous studies have demonstrated the applicability of biomass-derived carbon materials for the adsorption of dyes using pinecone-derived activated carbon [5] and the removal of tetracycline using sunflower husk-derived porous biochar [6]. These findings highlight the importance of evaluating both textural and physicochemical properties when assessing the potential environmental applications of activated carbons [4,5,6].

One of the fundamental stages in activated carbon production is carbonisation, which involves the thermal decomposition of organic material under oxygen-deficient conditions. This process is accompanied by the release of volatile products, an increase in the degree of carbonisation of the solid residue and the formation of a primary porous structure. Carbonisation temperature affects the decomposition of biomass components, the distribution of process products and the properties of the resulting char. Increasing the temperature generally promotes a reduction in volatile matter content and an increase in fixed carbon content, while simultaneously decreasing the mass yield of the solid product [7,8]. The influence of carbonisation temperature on the properties of carbonaceous materials has been demonstrated for both woody biomass and agricultural residues. Chowdhury et al. [9] described changes in the physicochemical properties of biochar produced from durian wood sawdust at different slow-pyrolysis temperatures. Liu et al. [10], in turn, highlighted the importance of carbonisation temperature in shaping the properties of biochars obtained from crop residues. These studies support the consideration of temperature as a parameter affecting not only the degree of carbonisation, but also the development of the material’s surface and its chemical properties. However, the direction and magnitude of these changes depend on the type of precursor; therefore, conditions beneficial for one feedstock cannot be directly transferred to other types of biomass [9,10].

Carbonisation does not always provide a sufficient degree of porosity development to obtain a material with the desired adsorption properties. Therefore, the char is subjected to activation, which opens and develops the pore network. Physical activation may be carried out using, among other agents, steam or carbon dioxide, which react with the carbon matrix at elevated temperatures. Controlled partial gasification of carbon enables the formation of new pores and the widening of existing ones. The importance of the activating agent and process conditions has been demonstrated in studies of carbons produced from olive stones and walnut shells [11,12]. Steam activation is particularly attractive because it enables the development of porosity without impregnating the feedstock with chemical activating agents. This reduces the demand for reagents used, among others, in potassium hydroxide, phosphoric acid or zinc chloride activation, as well as the need to remove their residues from the final product. However, the process requires an adequate energy supply and precise control of the degree of material gasification [3,13]. Increasing the activation temperature may intensify pore development, but it may also increase carbon mass loss. Excessive process severity may result in pore widening and coalescence; consequently, a further increase in temperature does not necessarily lead to a proportional improvement in adsorption properties. The selection of activation conditions should therefore account for both the development of the porous structure and the yield of sorbent production [11,12].

The quality assessment of biomass-derived activated carbons should also include ash content, acid–base properties and the functional parameters of the material. Mineral components present in the precursor are partly retained in the solid product, and their relative proportion may increase as a result of the removal of organic matter during thermal treatment. Differences in biomass composition and in the carbonisation and activation methods consequently lead to materials with different textural and chemical properties [4,10]. This justifies the combined analysis of sorption and physicochemical indicators, which enables a more comprehensive assessment of the suitability of a given feedstock for activated carbon production.

Potentially important precursors from the agri-food sector include residues from sugar beet processing, sunflower husks, apple pomace, straw and corn cobs. These materials represent different types of biomass, providing an opportunity to compare their behaviour during thermal treatment. Studies on sunflower husks have confirmed the possibility of producing porous materials suitable for the adsorption of contaminants from water [6]. Barszcz et al. [14] investigated the effect of pyrolysis conditions on the structure of biochar obtained from apple waste, whereas Pallarés et al. [15] described the production and characterisation of activated carbon from barley straw. These studies indicate the potential of individual feedstocks while also supporting comparative research conducted under common processing variants and using uniform evaluation criteria. In this context, it is important to determine how different precursors respond to changes in thermal conditions and whether process intensification provides similar benefits for each of them. Comparing several types of biomass makes it possible to identify feedstocks for which improved sorption properties are accompanied by a favourable combination of other material parameters. Such an approach provides a basis for selecting production conditions for sorbents while accounting for the specific characteristics of the available feedstock.

The aim of this study was to assess the relationship between two-stage thermal treatment conditions and the physicochemical and sorption properties of activated carbons produced from five agricultural and agri-food feedstocks: sugar beet pulp, sunflower husks, apple pomace, straw and corn cobs. Three sequences of carbonisation and subsequent steam-activation temperatures were compared: 400/600 °C, 500/700 °C and 600/850 °C. Selected feedstocks were pelletised using beet molasses as a binder. The originality of the study lies in the integrated assessment of the different responses of these feedstock variants to increasing thermal treatment severity, carried out according to a common experimental scheme and using uniform analytical criteria. Relating the iodine number and BET specific surface area to process yields, ash content and other physicochemical parameters made it possible to determine the extent to which improvements in adsorption properties were accompanied by adverse changes in process yield and product quality. This approach enabled the most favourable combinations of feedstock and processing conditions to be identified, while also revealing the limitations associated with process intensification. The results provide a basis for targeted optimisation of sorbent production from waste biomass.

## 2. Materials and Methods

### 2.1. Characterization of Raw Materials and Binder

**Figure 1:**
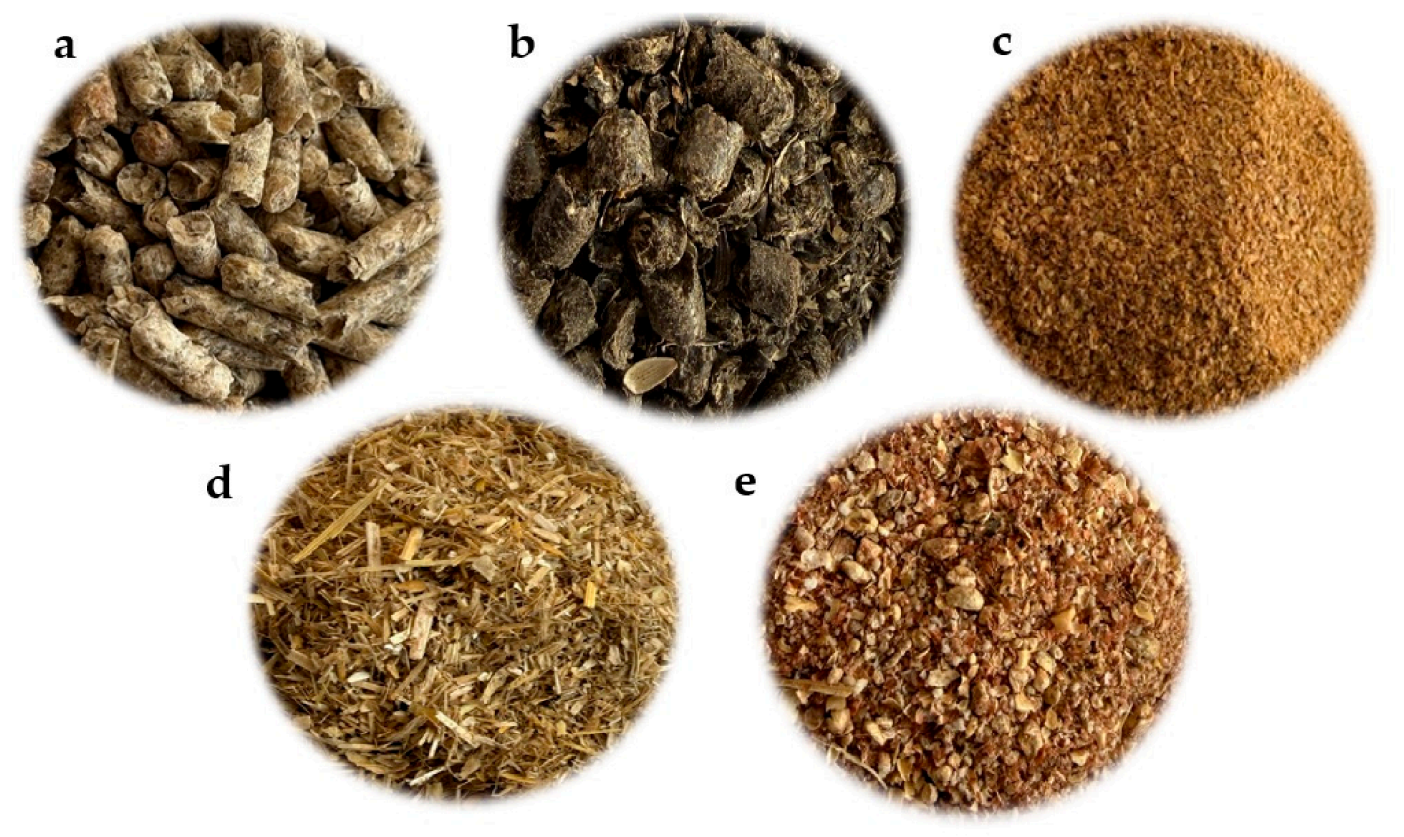
Raw materials used to produce carbon materials: (**a**) granulated sugar beet pulp; (**b**) sunflower husk pellets; (**c**) shredded apple pomace; (**d**) shredded straw; (**e**) shredded corn cobs.

Five agricultural and agri-food raw materials (Figure 1) were used to produce the carbon materials: granulated sugar beet pulp, sunflower husk pellets, apple pomace, straw, and corn cobs. The granulated sugar beet pulp was commercially available as feed material, whereas the sunflower husk pellets were commercially available as biomass fuel. Apple pomace, straw, and corn cobs were used in shredded form and pelletized with the addition of beet molasses prior to thermal treatment. No additional binder was added to the granulated sugar beet pulp or sunflower husk pellets. Beet molasses, a by-product of sugar production, served as a binder enabling the formation of shredded raw materials. Before use, its refractometric extract, expressed in Brix, pH, and dynamic viscosity, was determined. The biomass feedstocks were characterized in terms of moisture, ash, and volatile matter content. Analyses of the raw materials, binder, and carbonaceous materials were conducted at the Research and Development Department of Grand Activated.

### 2.2. Preparation of Raw Materials

The preliminary preparation of the raw materials involved drying them in a laboratory dryer (BINDER GmbH, Tuttlingen, Germany) at 120 °C for 3 h. Apple pomace, straw, and corn cobs, which were used in ground form, were mixed with beet molasses. For each mixture, 2 kg of biomass was weighed. The molasses was heated to 50 °C and gradually added to the raw material placed in a conditioning mixer. Mixing continued until a homogeneous paste was obtained that retained its cohesion after gentle compression. The amount of binder was varied depending on the type of raw material (Table 1).

**Figure 2:**
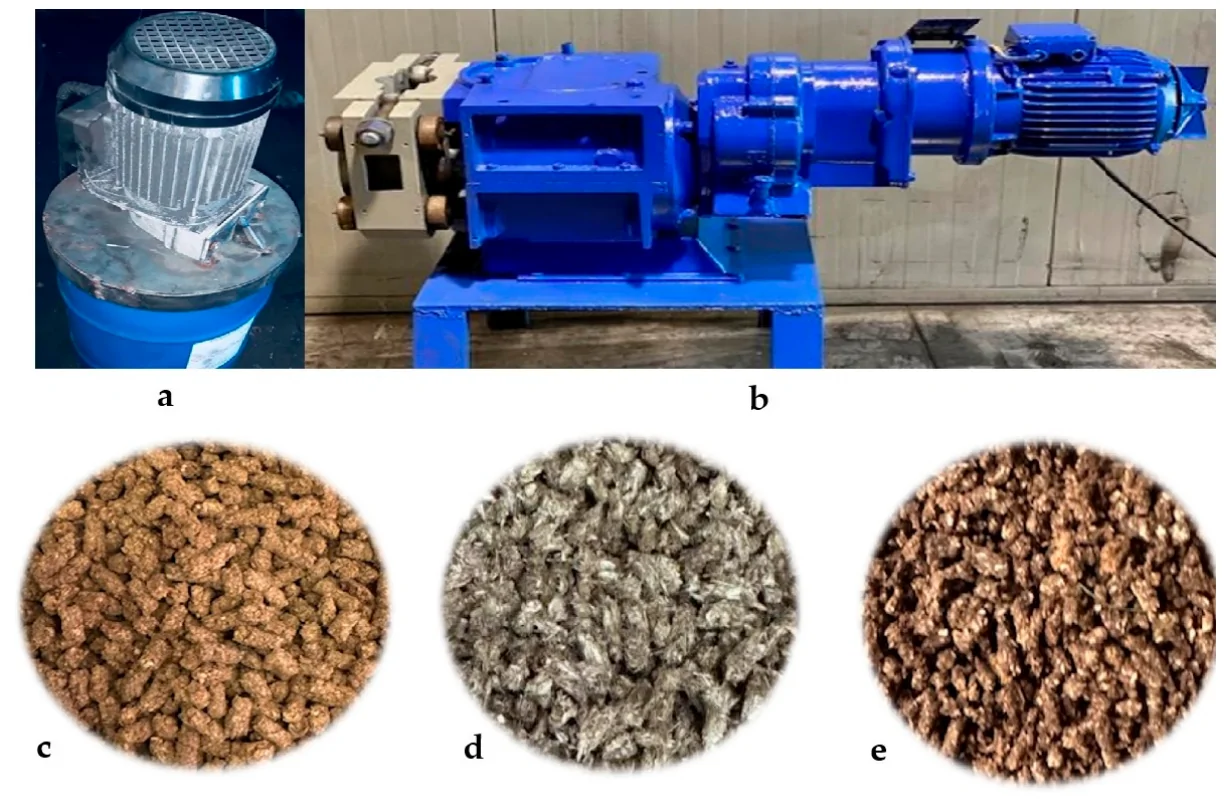
Preparation of granulated precursors with the addition of beet molasses: (**a**) conditioning mixer; (**b**) laboratory pelletizer equipped with a die with 4 mm diameter openings; granules obtained from (**c**) apple pomace, (**d**) straw, and (**e**) corn cobs.

The prepared mixtures were fed in batches into a laboratory pelletizer (custom-built device manufactured in-house by Grand Activated Sp. z o.o., Hajnówka, Poland) equipped with a die featuring 4.0 mm diameter openings. The material was formed by mechanically forcing the mixture through the die openings. The resulting granules were dried at 120 °C for 24 h and subsequently subjected to carbonization. The equipment used for raw material preparation, namely the mixer and pelletizer, together with the materials obtained after pelletization, are presented in Figure 2.

### 2.3. Carbonization and Steam Activation

Carbon materials were produced in a laboratory tube furnace model FCF 22SHM (Czylok Sp. z o.o., Jastrzębie-Zdrój, Poland) using a process comprising carbonization followed by physical steam activation. For each raw material, three corresponding temperature combinations were applied for the two stages (Table 2).

**Figure 3:**
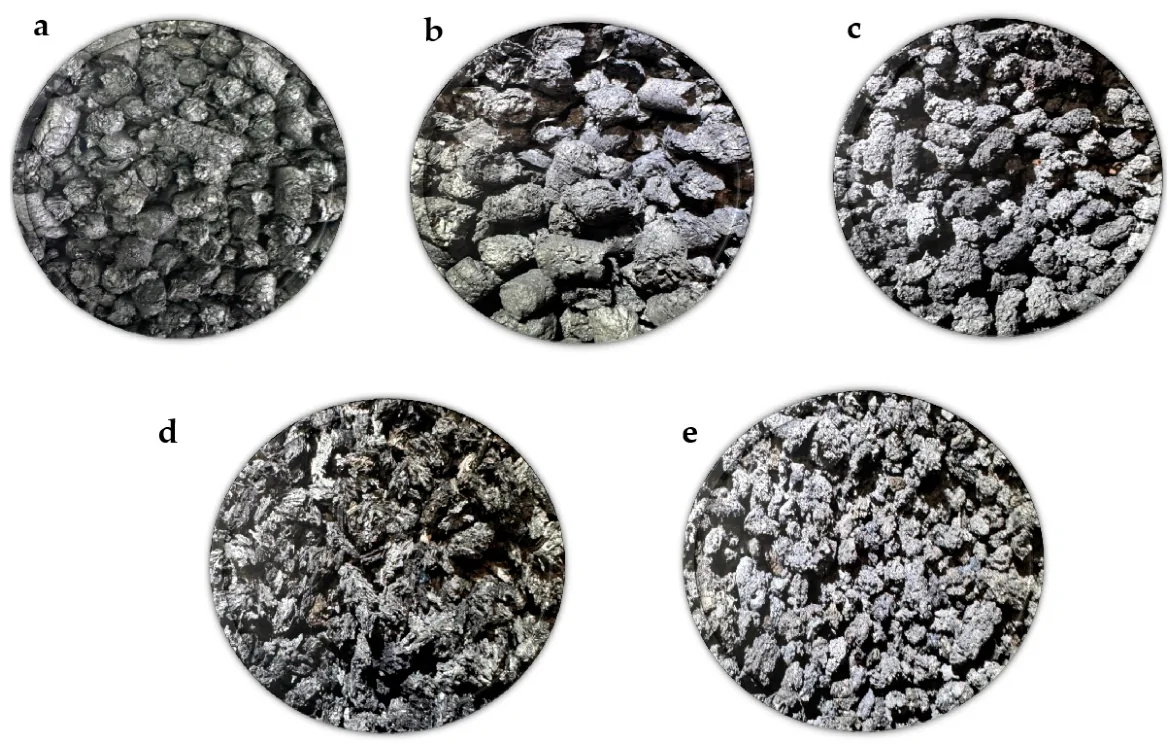
Carbon materials obtained after carbonization and steam activation of: (**a**) granulated sugar beet pulp; (**b**) sunflower husk pellets; (**c**) apple pomace; (**d**) straw; and (**e**) corn cobs.

Carbonization was conducted under oxygen-limited conditions. The heating program was initiated at a setpoint of 200 °C, after which the temperature gradually increased in 50 °C increments until the target temperature of 400, 500, or 600 °C was reached. The material was held at the final temperature for 60 min. Each carbonization variant was performed in triplicate to obtain material for analysis and subsequent activation. The carbonizates obtained at 400, 500, and 600 °C were activated with steam at 600, 700, and 850 °C, respectively. Activation was conducted for 60 min in a tube furnace, with a total of 190 mL of water supplied to generate steam during the activation process. Each activation variant was performed in quadruplicate to obtain enough material for physicochemical analyses. After completion of the thermal treatment, the materials were cooled and ground using a PULVERISETTE 2 mortar mill (FRITSCH GmbH, Idar-Oberstein, Germany) to a particle size below 0.12 mm. Further sample preparation was adjusted to the requirements of the individual analytical determinations. The material obtained from straw under the 600/850 °C treatment variant was excluded from the analytical characterization due to the almost complete disappearance of the carbonaceous material, documented experimentally as ashing. The carbonization and activation temperatures were varied in combination; therefore, they were not treated as independently varied experimental factors. Comparisons among the raw materials accounted for differences in their preparation procedures and the varying amounts of beet molasses used. The carbon materials obtained from the five biomass feedstocks after carbonization and subsequent steam activation are presented in Figure 3.

### 2.4. Determination of Carbonization and Steam Activation Yields

Carbonization and activation yields were determined separately from the mass of the feedstock introduced into each stage and the mass of the resulting product. The percentage mass loss and process yield were calculated using the following equations:

(1) $$m\;=\;(m_{1}\;-\;m_{2})\;/\;m_{1}\;\times \;100\%\;$$

*where*

*Δm*—percentage mass loss;
*m*_1_—mass of the feedstock charged into the furnace (g);
*m*_2_—mass of the product obtained (g).

(2) $$W=(m_{2}\;/\;m_{1})\times 100\%$$

*where*

*W*—percentage process yield.

The mean yields of the carbonization and steam activation processes were determined for all investigated biomass feedstocks and process temperatures. The carbonization yields obtained at 400, 500, and 600 °C are summarized in Table 3, whereas the corresponding activation yields at 600, 700, and 850 °C are presented in Table 4. The results enable comparison of the influence of the applied thermal treatment conditions and the type of biomass feedstock on the mass yield of the obtained carbonaceous materials.

### 2.5. Physicochemical Characterization

The raw materials were analyzed for moisture, ash, and volatile matter contents. The carbon chars were characterized based on iodine number, bulk density, ash content, volatile matter content, and the calculated fixed carbon content. For the activation products, the iodine number, specific BET surface area, bulk density, ash content, volatile matter content, moisture content, and pH of the aqueous extract were determined.

The iodine number was determined by titration according to the procedure specified in PN-83/C-97555.04 [16] and the experimental documentation. Approximately 1 g of the laboratory sample was collected and ground until the entire material passed through a sieve with a mesh size of 0.071 mm. The material was dried at 110 ± 5 °C to constant mass. A 0.2000 g portion, weighed with an accuracy of 0.0002 g, was transferred to a 100 mL flask. Subsequently, 4 mL of 5% HCl solution and 20 mL of 0.2 mol·L^−1^ iodine solution were added. The flask was stopped and shaken for 4 min using a DOS-20 shaker (ELMI Ltd., Riga, Latvia). The contents were filtered through a soft quantitative filter, and the residue retained on the filter was washed with 50 mL of distilled water. The entire filtrate, together with the washing water, was titrated with 0.1 mol·L^−1^ sodium thiosulfate solution using a 1% starch solution as the indicator. A blank determination was performed in parallel using the same amounts of reagents. The coefficient 126.90 results from the stoichiometry of the reaction between iodine and thiosulfate.

*LJ* = (*Vᵦ − V_s_*) · *c_t_* · 126.90/*m* (3)

*where*

*LJ*—iodine number (mg·g^−1^);
*Vᵦ* and *V****_s_***—titrant volumes consumed for the blank and sample titrations, respectively (mL);
*c_t_*—molar concentration of sodium thiosulfate (mol·L^−1^);
*m*—mass of the dry test portion (g).

The specific surface area of the activation products was determined by the BET method using nitrogen adsorption in a SORPTY 1750 analyzer (Carlo Erba Instruments, Milan, Italy), following the procedure specified in the PN-ISO 9277:2000 standard [17]. The sample was dried at 110 °C to constant mass. Approximately 1 g of material was weighed with an accuracy of 0.0002 g, transferred to the measurement burette, and degassed under vacuum. Measurements were performed using liquid nitrogen as the cooling medium. The results were expressed in m^2^·g^−1^.

For comparative purposes, the adsorption-related physicochemical properties of selected commercial activated carbons were obtained from publicly available manufacturers’ technical datasheets. The comparison was limited to BET specific surface area and iodine number, which were also determined for the investigated biomass-derived materials. The commercial reference values were used exclusively for contextual benchmarking and did not represent experimental measurements performed within the present study.

Moisture content was determined by the drying-oven method according to PN-84/C-97555.09. Approximately 2 g of homogenized material was collected and evenly distributed in a previously dried and weighed weighing vessel. The sample was dried at 105–110 °C for 2 h. The vessel cooled in air for 3 min, then placed in a desiccator until reaching room temperature, and weighed. Control drying continued in 30 min intervals until the difference between two consecutive weighings was less than 0.001 g.

*M* = (*m*_1_ − *m*_2_) · 100/(*m*_1_ − *m*_0_) (4)

*where*

*M*—moisture content (%);
*m*_0_—mass of the empty weighing vessel (g);
*m*_1_—mass of the vessel with the sample before drying (g);
*m*_2_—mass of the vessel with the sample after drying to constant mass (g).

All masses are expressed in grams.

Ash content was determined according to PN-84/C-97555.08 [18]. The material was ground to a fraction passing through a sieve with a mesh size of 0.200 mm, homogenized, and dried at 105–110 °C to constant mass. A 2.0 ± 0.1 g portion, weighed with an accuracy of 0.0002 g, was placed in a porcelain crucible previously ignited to constant mass. The crucible was placed in a cold muffle furnace, heated to 815 ± 15 °C, and maintained at this temperature for 60 min. The crucible was cooled in air for 5 min and then in a desiccator. Control ignition continued in 20 min intervals until the difference between consecutive weighings did not exceed 0.001 g.

*Aᵈ* = (*mₐ* − *m*_0_) · 100/(*mᵈ* − *m*_0_) (5)

*where*

*A^d^*—ash content on a dry-mass basis (%);
*m*_0_—mass of the empty crucible (g);
*m^d^—*mass of the crucible with the dry sample (g);
*mₐ—*mass of the crucible with the ash residue (g).

The masses are expressed in grams.

Volatile matter content was determined using the procedure described in PN-EN 1860-2:2006 [19], adapted to the carbonaceous materials investigated. Approximately 1 g of sample was weighed with an accuracy of 0.1 mg into a previously heated and cooled crucible. The sample was dried without the lid at 105–110 °C for 1 h, cooled in a desiccator, and weighed. The crucible was then closed with a properly fitted lid and placed in an FCF 22SHM muffle furnace preheated to 900 ± 10 °C. Heating was conducted for exactly 7 min, after which the crucible was cooled in a desiccator and weighed.

*Vᵈ* = (*mᵈ* − *mᵣ*) · 100/(*mᵈ* − *m*_0_) (6)

*where*

*Vᵈ—*volatile matter content on a dry-mass basis (%);
*m*_0_—mass of the empty crucible with the lid (g);
*mᵈ—*mass of the crucible with the lid and dry sample before heating (g);
*mᵣ*—mass of the same set after heating at 900 °C (g).

The masses are expressed in grams.

*FCᵈ* = 100 − *Aᵈ* − *Vᵈ* (7)

The fixed carbon content in the carbonizates (*FCᵈ*, %) was calculated by difference based on the ash and volatile matter contents on a dry-mass basis. This parameter represents a proximate-analysis quantity and is distinct from elemental carbon content determined by elemental analysis.

Tapped bulk density was determined according to the procedure specified in PN-EN 12903:2009 using a STAV 2003 volumeter (J. Engelsmann AG, Ludwigshafen, Germany). The sample was dried at 150 ± 10 °C to constant mass. The material was introduced through a funnel into a graduated cylinder at a constant rate of approximately 1 mL·s^−1^ until a volume of 100 mL was reached. The mass of the material was then determined with an accuracy of 0.1 g.

*ρᵦ* = *m*/*V* (8)

*where*

*ρᵦ*—bulk density (g·L^−1^);
*m*—mass of the dry sample (g);
*V*—volume occupied by the sample, equal to 0.100 L.

The pH of the aqueous extract was determined potentiometrically according to PN-85/C-97555.10. A 5.0 g portion of material was weighed with an accuracy of 0.1 g, transferred to a 100 mL flask, and mixed with 50 mL of distilled water. The material-to-water ratio was 1:10 (g:mL). The suspension was mixed for 5 min and then left to allow separation of the solid phase. Approximately 20 mL of the liquid was decanted, and the pH was measured using a CPC-411 pH meter (ELMETRON, Zabrze, Poland). The reported value was the mean of two parallel determinations, for which the difference did not exceed 0.3 pH units.

### 2.6. Characterization of Sugar Beet Molasses

The binder was characterized following the procedures identified in the experimental documentation PN-76/R-64772 [20]. The Brix value was measured using a DR201-95 refractometer(A. KRÜSS Optronic GmbH, Hamburg, Germany). After calibration, approximately 1 mL of thoroughly mixed molasses was placed on the prism, and the reading was taken according to the instrument manufacturer’s instructions. The result was reported as the mean of duplicate measurements. The Brix value was interpreted as a refractometric indicator of soluble solids content rather than a selective measurement of sugar content.

For pH determination, 250.0 ± 0.5 g of molasses was weighed, and approximately 250 mL of hot distilled water was added with stirring. After dissolution, the sample was cooled in a water bath to approximately 20 °C, and distilled water was added to obtain a total mass of 500.0 ± 0.5 g. The pH was measured using a CPC-411 meter (ELMETRON Sp.j., Zabrze, Poland) at 20.0 ± 0.5 °C after rinsing the electrode with the prepared solution. The result was reported as the mean of duplicate determinations.

Dynamic viscosity was determined using a Reotest 2 viscometer (VEB MLW Prüfgeräte-Werk Medingen, Medingen, Germany). equipped with a type S measuring system. A 22 mL aliquot of homogenized molasses was transferred to the measuring vessel. Measurements were performed at 20, 30, 40, 50, and 55 °C. Viscosity was calculated from the instrument readings using the coefficients corresponding to the selected measurement range. Results were expressed in mPa·s, which are numerically equivalent to cP.

### 2.7. Scanning Electron Microscopy and Energy-Dispersive X-Ray Spectroscopy

The surface morphology and elemental composition of selected activated carbons were examined using a Scios 2 DualBeam scanning electron microscope (SEM/FIB, Thermo Fisher Scientific, Waltham, MA, USA). equipped with an energy-dispersive X-ray spectroscopy (EDS) system. Prior to examination, the samples were coated with an approximately 5 nm thick conductive gold layer using a Leica EM ACE200 sputter coater (Leica Microsystems GmbH, Wetzlar, Germany).

SEM observations were performed under high-vacuum conditions at an accelerating voltage of 2 kV, using an Everhart–Thornley secondary electron detector (ETD) (ETD; Thermo Fisher Scientific, Waltham, MA, USA) and a spot size of 5.0. Micrographs were acquired at different magnifications to assess the overall particle morphology and local surface features.

Elemental analysis and elemental distribution mapping were performed using EDS at an accelerating voltage of 10 kV. The electron beam conditions were adjusted to maintain a detector dead time of approximately 10–12%. The obtained EDS spectra were used to identify the elements present in the investigated regions, while elemental maps were acquired to assess their spatial distribution.

### 2.8. Statistical Analysis

Statistical analysis was performed to evaluate the variability and repeatability of the physicochemical measurements obtained for the investigated biomass feedstocks, carbonizates, and activated carbons. Representative samples were collected from the materials obtained at the individual stages of the experimental procedure and homogenized prior to analysis.

For each biomass feedstock, moisture content, ash content, and volatile matter content were determined in triplicate (*n* = 3). In the case of carbonizates obtained at 400, 500, and 600 °C, triplicate analytical determinations included ash content, volatile matter content, and bulk density. For activated carbons produced under the three combined carbonization and steam activation temperature sequences (400/600, 500/700, and 600/850 °C), moisture content, ash content, volatile matter content, and bulk density were determined in triplicate for each investigated material formulation. The results of the physicochemical analyses were expressed as the arithmetic mean and standard deviation (mean ± SD). The arithmetic mean was calculated using Equation (9):

(9) $$\bar{x}=\frac{1}{n}\sum_{i=1}^{n}x_{i}$$

*where*

$\bar{x}$ is the arithmetic mean.
*x_i_* is the individual analytical measurement, and *n* is the number of determinations (*n* = 3).

The sample standard deviation was calculated according to Equation (10):

(10) $$\mathrm{SD}=\sqrt{\frac{\sum_{i=1}^{n}{\left(x_{i}\;-\;\bar{x}\right)}^{2}}{n\;-\;1}}$$

*where*

*SD* denotes the sample standard deviation; *x_i_*, $\bar{x}$, and *n* are defined as above.

The standard deviation was used to quantify the dispersion of individual measurement results around the arithmetic mean and to assess analytical repeatability.

The fixed carbon content (*FC*) of the carbonizates was calculated by difference from the measured ash content (*A*) and volatile matter content (*VM*), as described in Section 2.5. Since fixed carbon was calculated rather than a directly measured parameter, its uncertainty was estimated by propagating the standard deviations of ash and volatile matter contents, assuming that the corresponding measurement errors were uncorrelated. The propagated standard deviation was calculated using Equation (11):

(11) $$\mathrm{SD}(\mathrm{FC})=\sqrt{{\mathrm{SD}(A)}^{2}+{\mathrm{SD}(\mathrm{VM})}^{2}}$$

where

*SD(FC)* estimated standard deviation of the calculated fixed carbon content;
*SD(A)* standard deviation of ash content;
*SD(VM)* standard deviation of volatile matter content.

The calculated mean values and their corresponding standard deviations are presented in the tables as mean ± *SD*. Where applicable, graphical representations include error bars corresponding to ±1 SD. For calculated fixed carbon content, the error bars represent propagated uncertainty based on the variability of the contributing analytical parameters.

The triplicate physicochemical determinations were treated as analytical replicates and distinguished from the repeated thermal processing experiments described in Section 2.3. Therefore, the reported analytical standard deviations characterize the variability of the measurements and should not be interpreted as direct estimates of between-batch process variability.

The iodine number, BET specific surface area, carbonization and activation yields, and aqueous extract pH were evaluated descriptively where sufficient replicate-level data for calculating standard deviations were unavailable. No unsupported uncertainty estimates were assigned to these parameters.

The results obtained were analyzed descriptively to compare the investigated biomass formulations and thermal treatment sequences. No inferential statistical tests were applied, and no claims of statistical significance were made.

Selected graphical representations of the experimental results were prepared using ython (version 3.13.5) and the Matplotlib library (version 3.10.8). ChatGPT 5.6 (OpenAI) was used to assist in preparing the plotting scripts and organizing the graphical presentation of the data. The numerical values and uncertainty estimates presented in the figures were derived from the experimental dataset and the statistical calculations described above.

## 3. Results and Discussion

The properties of activated carbons derived from biowaste vary considerably depending on the biomass feedstock and the pyrolysis and activation conditions. Key characteristics of biomass-derived carbonaceous materials include chemical composition, structural stability, specific surface area, and pore development. Their chemical composition is influenced by both precursor properties and processing parameters, particularly temperature and residence time. The biomass feedstocks and resulting carbonaceous materials were characterized at successive preparation stages to evaluate their properties at each selected carbonization and activation temperature. The analytical methods and assessment criteria were consistent with those used in commercial activated carbon production. Following pyrolysis, the chars were analyzed for iodine number, ash content, volatile matter content, and bulk density, while fixed carbon content was calculated. Following activation, the resulting activated carbons were characterized in terms of iodine number, BET specific surface area, ash content, volatile matter content, moisture content, bulk density, and aqueous extract pH.

### 3.1. Biowaste Feedstocks for Activated Carbon Production

The five biomass feedstocks were characterized by moisture, ash, and volatile matter contents (Table 5). The results revealed relatively similar volatile matter contents but pronounced differences in ash content, indicating variation in the mineral fraction of the materials selected for carbonization and subsequent steam activation.

**Figure 4:**
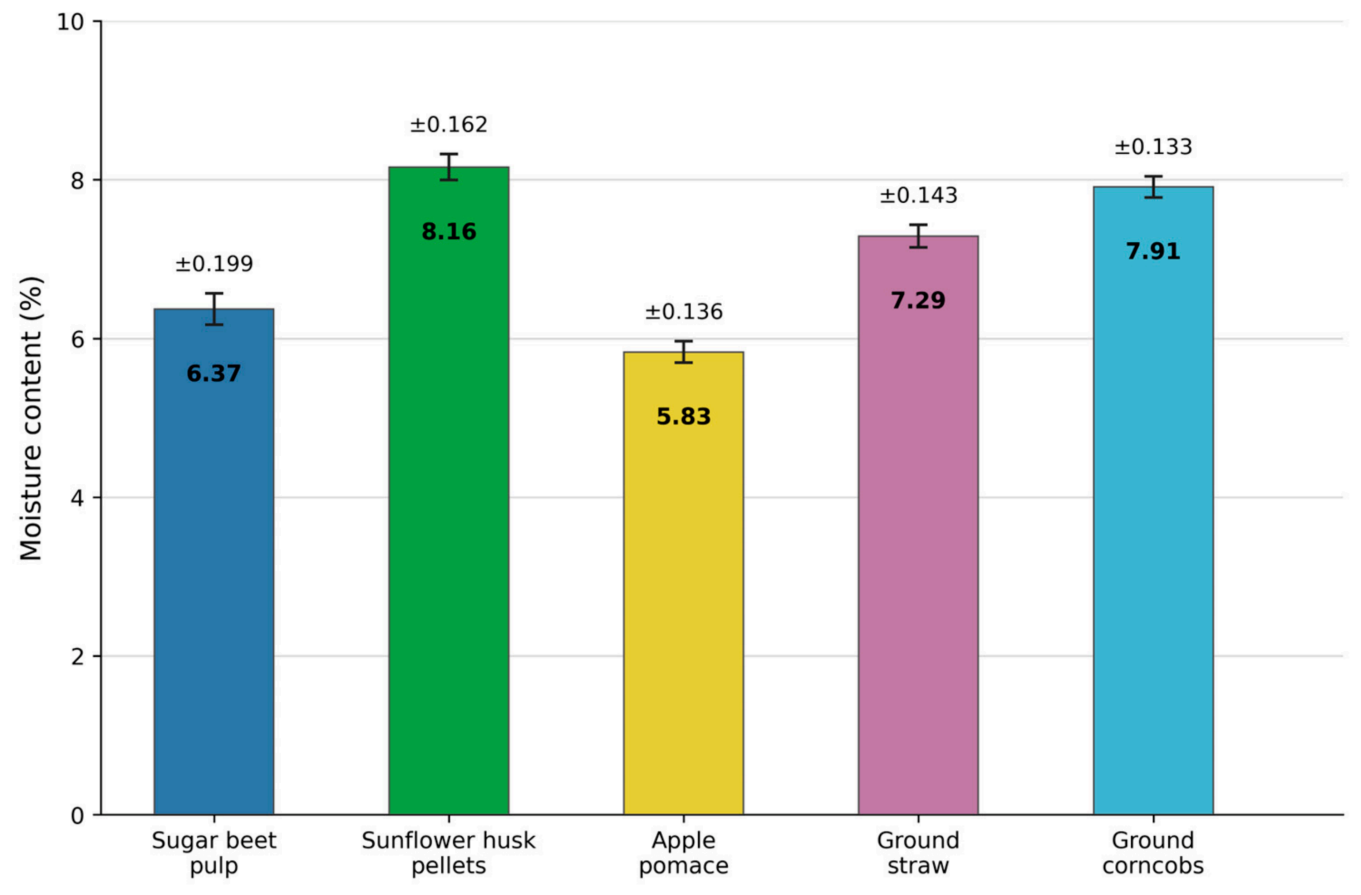
Moisture content of the biomass feedstocks used for activated carbon production. Bars represent mean values, and error bars indicate ±1 standard deviation (SD, *n* = 3). Mean values are displayed inside the bars, while SD values are provided above the error bars.

Moisture content ranged from 5.83% for apple pomace to 8.16% for sunflower husk pellets, corresponding to a difference of 2.33 percentage points (Figure 4). Intermediate values were recorded for sugar beet pulp (6.37%), ground straw (7.29%), and ground corn cobs (7.91%). These values characterize the prepared feedstocks at the time of analysis rather than the freshly generated agricultural or processing residues.

**Figure 5:**
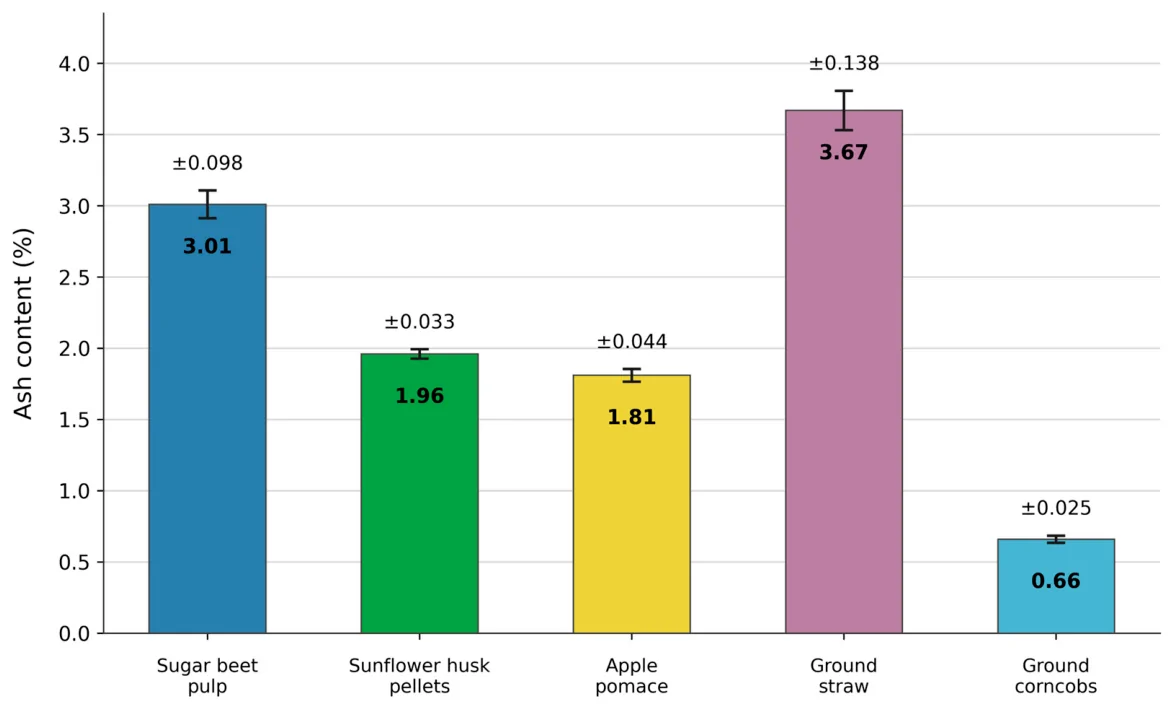
Ash content of the biomass feedstocks used for activated carbon production. Bars represent mean values, and error bars indicate ±1 standard deviation (SD, *n* = 3). Mean values are displayed inside the bars, while SD values are provided above the error bars.

Ash content showed the greatest relative variation among the measured parameters (Figure 5). Ground corn cobs exhibited the lowest value (0.66%), followed by apple pomace (1.81%) and sunflower husk pellets (1.96%). Higher values were recorded for sugar beet pulp (3.01%) and ground straw (3.67%). Thus, the ash content of straw was approximately 5.6 times that of corn cobs. These differences provide a baseline for interpreting the mineral residue contents of the resulting carbonaceous materials. However, initial ash content alone does not establish precursor suitability, which also depends on the response of each material to carbonization and activation.

**Figure 6:**
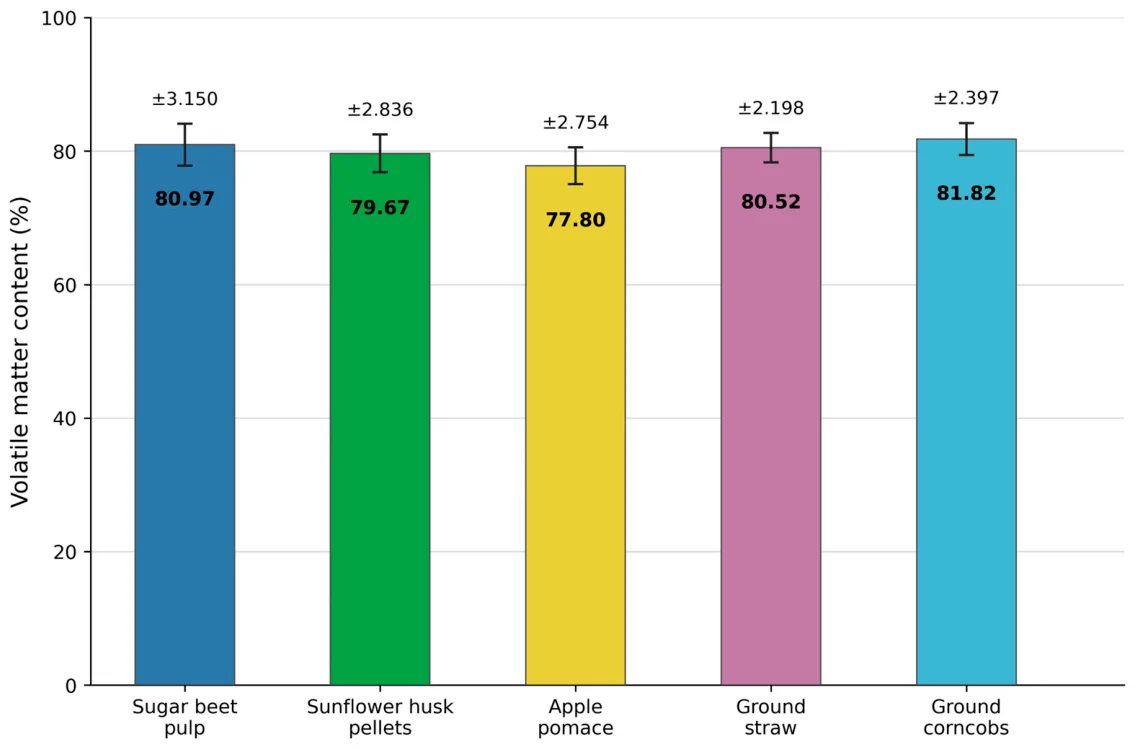
Volatile matter content of the biomass feedstocks used for activated carbon production. Bars represent mean values, and error bars indicate ±1 standard deviation (SD, *n* = 3). Mean values are displayed inside the bars, while SD values are provided above the error bars.

Volatile matter content varied within a comparatively narrow range of 77.80–81.82% (Figure 6). The highest value was obtained for ground corn cobs, followed by sugar beet pulp (80.97%), ground straw (80.52%), and sunflower husk pellets (79.67%), whereas apple pomace exhibited the lowest value. The difference between the maximum and minimum values was 4.02 percentage points. The consistently high values indicate a substantial fraction released under the conditions of the volatile matter determination; they do not, however, directly predict carbonization yield or the extent of pore development during subsequent activation.

Overall, ground corn cobs combined the lowest initial ash content with the highest volatile matter content, whereas straw contained the largest mineral residue fraction. Apple pomace and sunflower husk pellets exhibited intermediate ash contents below 2%. These results establish the initial physicochemical characteristics against which subsequent changes in the chars and activated carbons can be evaluated. For apple pomace, straw, and corn cobs, interpretation of the final material properties must additionally account for the molasses introduced during granulation, as the values reported in Table 5 and Figure 4, Figure 5 and Figure 6 characterize the biomass feedstocks rather than the biomass–binder mixtures.

### 3.2. Results of Binder Characterization

Sugar beet molasses was used as a binder during the granulation of apple pomace, ground straw, and ground corn cobs. Its characterization included moisture content, refractometric soluble solids content, pH, and dynamic viscosity. The reported values are presented in Table 6.

The molasses had a moisture content of 22.1% and a refractometric reading of 79.9 Brix, characterizing a concentrated binder formulation. These measurements describe different analytical properties and should not be treated as complementary mass fractions. In particular, the Brix reading cannot be interpreted as a directly measured sugar concentration, because the refractive response of molasses also reflects dissolved non-sugar constituents.

The pH of the prepared molasses solution was 6.2, indicating a mildly acidic reaction under the measurement conditions. This value characterizes the diluted solution used in the analytical procedure and does not establish the pH of the undiluted binder or the surface chemistry of the carbonaceous products obtained after thermal treatment.

The reported dynamic viscosity was 446 mPa·s. Although this parameter is relevant to binder handling and mixing with ground biomass, the results include only one viscosity value, without an associated measurement temperature. Consequently, the temperature dependence of viscosity cannot be evaluated from the available dataset, despite measurements at several temperatures being described in the experimental procedure.

The measured properties provide a physicochemical description of the molasses introduced during feedstock preparation. However, they do not independently demonstrate an improvement in granule strength, carbonization yield, or adsorption performance. Because the binder was added to selected feedstocks at different dosages, the properties of the resulting carbonaceous materials should be interpreted in relation to the complete biomass–binder formulations.

### 3.3. Carbonization Yield and Char Properties

**Figure 7:**
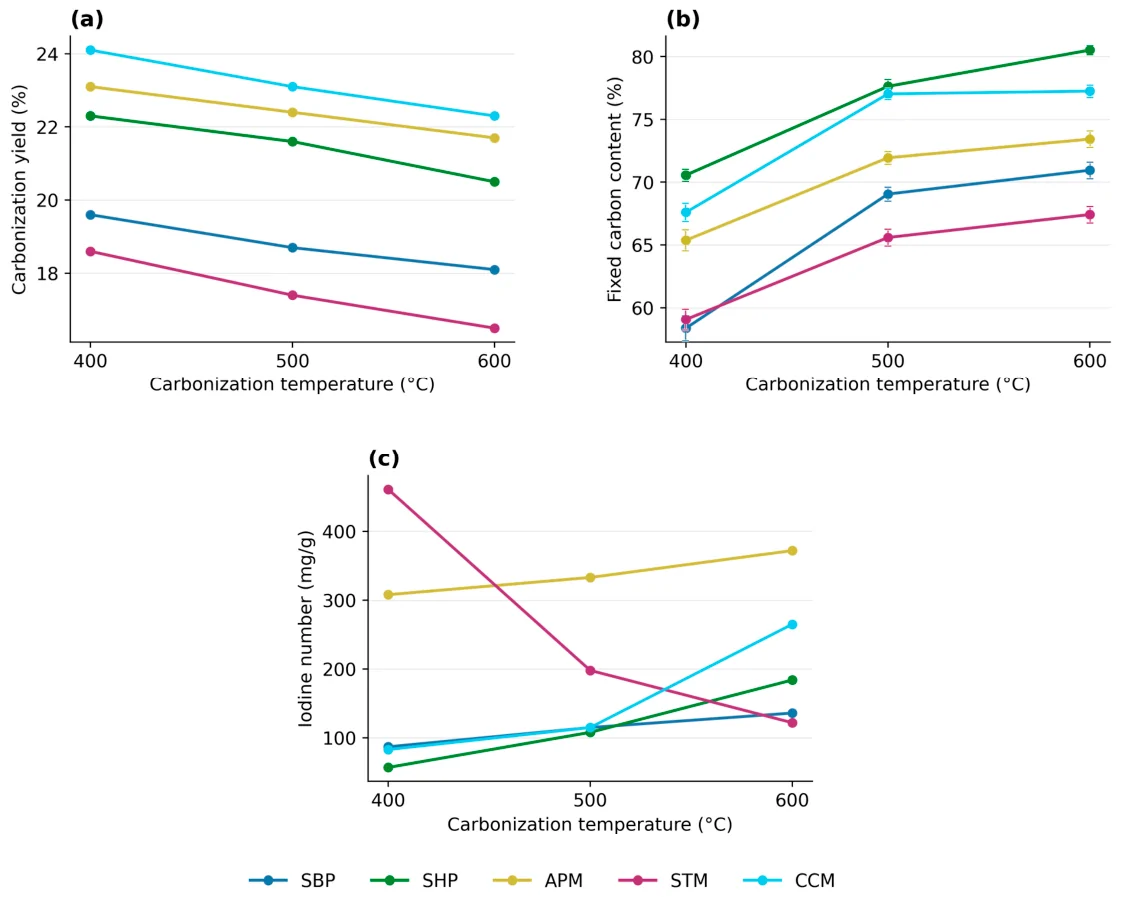
Effect of carbonization temperature on (**a**) mass yield, (**b**) calculated fixed carbon content, and (**c**) iodine number. Error bars in panel (**b**) represent propagated uncertainty calculated from the standard deviations of ash and volatile matter contents, assuming uncorrelated measurement errors. SBP: sugar beet pulp; SHP: sunflower husk pellets; APM: apple pomace with molasses; STM: straw with molasses; CCM: corn cobs with molasses.

Increasing the carbonization temperature from 400 to 600 °C reduced the mass yield of all five feedstock formulations (Figure 7a). The observed decrease in carbonization yield with increasing temperature can be associated with the progressive thermal decomposition of lignocellulosic constituents and the release of volatile degradation products. The relative contributions of cellulose, hemicellulose, lignin, and non-structural components differ among biomass precursors and may affect both the extent of mass loss and the characteristics of the residual carbon matrix. Similar temperature-dependent changes in solid yield and physicochemical properties have been reported for lignocellulosic biomass and agricultural residues [7,8,9,10]. However, the present results should be interpreted in relation to the investigated feedstock formulations, since the addition of molasses to selected materials may also influence their thermal conversion behaviour. The relative decrease ranged from 6.1% for APM to 11.3% for STM. CCM maintained the highest yield throughout the investigated range, whereas STM consistently exhibited the lowest values. These results show that the extent of mass loss depended on the feedstock formulation, even when the same temperature programme was applied.

The reduction in yield was accompanied by increasing fixed carbon content (Figure 7b) and decreasing volatile matter content (Table 7). SHP exhibited the highest fixed carbon content at each temperature, reaching 80.52% at 600 °C. The largest increase across the investigated interval occurred for SBP, amounting to 12.58 percentage points. Fixed carbon was calculated by difference from ash and volatile matter contents; its increase therefore describes changes in proximate composition rather than providing an independent measurement of structural ordering.

The iodine number increased with carbonization temperature for SBP, SHP, APM, and CCM, but decreased for STM (Figure 7c). The divergent trends in iodine number indicate that increasing carbonization temperature did not produce equivalent changes in adsorption-related properties across the investigated feedstocks. Thermal decomposition may facilitate the release of volatile compounds and improve access to adsorption sites within the resulting carbonaceous structure. Nevertheless, the extent of these changes depends on precursor composition and processing conditions. The particularly pronounced decrease in iodine number observed for straw may be related to differences in the evolution of its carbonaceous and mineral fractions; however, the available measurements do not establish the underlying mechanism. Therefore, additional textural and morphological characterization would be required to distinguish between changes in pore accessibility, structural alteration, and possible effects associated with the mineral components. The contrast was particularly pronounced between CCM, for which the iodine number increased approximately 3.2-fold, and STM, for which it decreased by 73.5%. Thus, increasing the degree of carbonization did not universally improve iodine adsorption. The mechanisms underlying the decline for STM cannot be resolved from proximate analysis and iodine adsorption measurements alone.

Ash content increased with carbonization temperature for all formulations, while volatile matter content decreased. STM had the highest ash content across the entire temperature range, reaching 20.25% at 600 °C. SHP maintained the lowest ash content at 500 and 600 °C and the lowest volatile matter content throughout the investigated range. These differences distinguish the composition of the chars entering the activation stage.

Bulk density decreased with carbonization temperature for SBP, SHP, STM, and CCM. APM showed a small increase from 202 to 206 g/L, demonstrating that this parameter did not follow a common trend across all formulations. Bulk density reflects particle properties and packing behaviour and cannot, by itself, establish changes in pore volume or skeletal density.

The contrasting iodine-number trends are therefore considered alongside, rather than inferred directly from, the changes in ash content and bulk density. In particular, the increase in fixed carbon content of STM occurred concurrently with a marked reduction in iodine adsorption, highlighting the need to evaluate compositional and adsorption indicators separately.

### 3.4. Changes in Adsorption Properties Following Steam Activation

**Figure 8:**
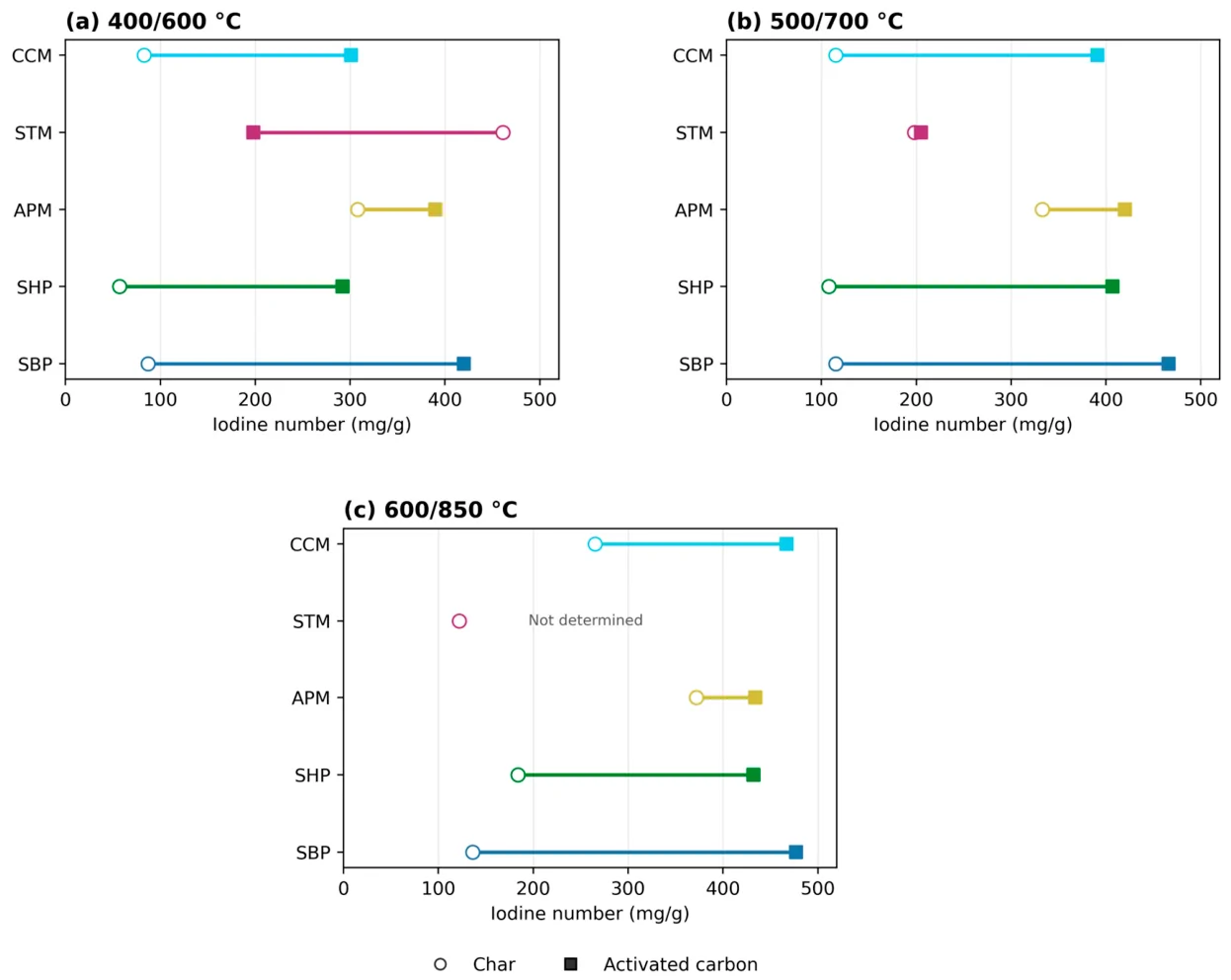
Iodine numbers of chars and their corresponding activated products for (**a**) 400/600 °C, (**b**) 500/700 °C, and (**c**) 600/850 °C carbonization/activation sequences. Open circles denote chars and filled squares denote activated products. Connecting segments indicate paired samples rather than intermediate measurements. SBP: sugar beet pulp; SHP: sunflower husk pellets; APM: apple pomace with molasses; STM: straw with molasses; CCM: corn cobs with molasses.

The effect of the activation stage was evaluated by comparing each activated product with its corresponding char (Table 8, Figure 8). The three investigated treatments comprised carbonization/activation temperature pairs of 400/600, 500/700, and 600/850 °C. Differences between the final products therefore represent the combined effects of these thermal sequences and cannot be attributed independently to activation temperature.

For SBP, SHP, APM, and CCM, activation increased the iodine number relative to the corresponding char in every sequence. The largest relative increases occurred in the 400/600 °C sequence for SHP and SBP, at approximately 5.1-fold and 4.8-fold, respectively. These large ratios partly reflect the low iodine numbers of the starting chars and should not be interpreted as evidence of the highest final adsorption performance.

STM exhibited a different response. In the 400/600 °C sequence, its iodine number decreased from 461 to 198 mg/g after activation. In the 500/700 °C sequence, only a small numerical increase was observed, from 198 to 205 mg/g. Following the 600/850 °C sequence, extensive loss of the carbonaceous fraction prevented characterization. The missing result is not equivalent to a measured iodine number or BET surface area of zero.

The increase in iodine number following steam activation observed for most of the investigated materials is consistent with the expected effects of partial gasification of the carbon matrix. During physical activation, steam reacts with carbon, potentially opening previously inaccessible regions and modifying the accessible pore network. Nevertheless, the relationship between activation conditions and iodine adsorption is not necessarily linear, because pore accessibility and the extent of carbon consumption may vary among different precursors. Previous investigations of physically activated biomass-derived carbons have demonstrated that the development of adsorption-related properties depends on both the initial char structure and the severity of the activation treatment [11,12,21,22]. The contrasting response of straw in the present study further highlights the importance of feedstock-dependent optimization.

**Figure 9:**
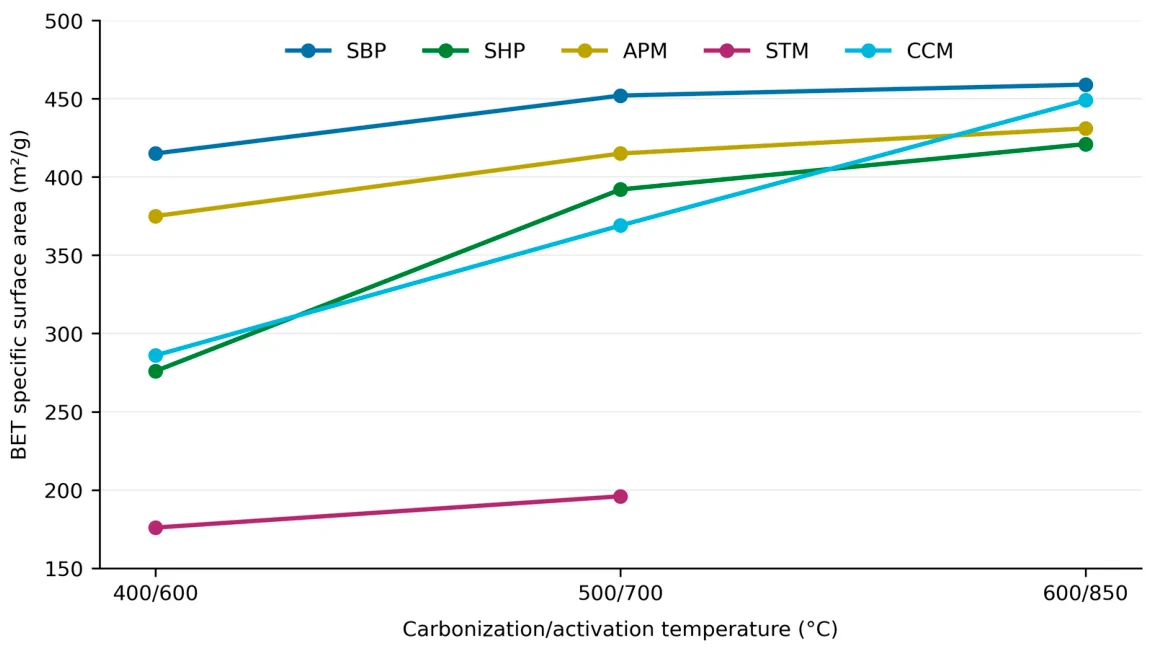
BET specific surface area of activated carbons obtained from different agricultural and agri-food waste materials under three combined carbonization/steam activation temperature sequences. Lines are included as visual guides connecting the experimental treatments. SBP: sugar beet pulp; SHP: sunflower husk pellets; APM: apple pomace with molasses; STM: straw with molasses; CCM: corn cobs with molasses. The result for STM at 600/850 °C is unavailable due to extensive loss of the carbonaceous material.

The BET surface area of the activated products increased across the thermal sequences for all formulations that remained available for analysis (Figure 9). The magnitude of the increase differed substantially: between 400/600 and 600/850 °C, BET surface area increased by 10.6% for SBP, 52.5% for SHP, 14.9% for APM, and 57.0% for CCM. Consequently, the benefit of more intensive thermal treatment was strongly formulation-dependent.

SBP exhibited the highest BET surface area in each sequence. However, increasing treatment severity from 500/700 to 600/850 °C produced an increment of only 1.5%, compared with 21.7% for CCM. APM also showed a limited increase over this interval, at 3.9%. These diminishing increments identify intermediate thermal conditions as candidates for further optimization, without establishing an energetic or economic optimum.

The limited additional increase in BET specific surface area observed for selected precursors at the highest treatment sequence may indicate diminishing benefits from further thermal intensification. Previous studies have shown that steam activation can promote the formation of new pores and the widening of existing pores, while excessive carbon consumption may eventually reduce the efficiency of surface area development. Zhang et al. [23], for example, demonstrated that the evolution of pore structure during steam activation depends on the treatment conditions and may involve both pore formation and pore widening. Nevertheless, the present study did not determine pore-size distributions or micropore volumes. Therefore, the relatively small BET increments observed for SBP and APM cannot be attributed unequivocally to a specific pore-development mechanism.

The complementary properties in Table 8 show that increased BET surface area was accompanied by increasing ash content. APM maintained the lowest ash content at the two higher treatment sequences, whereas STM exhibited the highest values in the two sequences for which measurements were possible. Moisture contents were between 1.99 and 3.01%. The aqueous extracts of APM were close to neutral, while those of the other activated products were alkaline. These pH values characterize the extracts and do not directly identify surface functional groups or the point of zero charge. The different responses of SBP and CCM to increasing thermal treatment severity were further examined by SEM analysis. The corresponding morphological observations are presented and discussed in Section 3.6.

The increase in ash content with increasing thermal treatment severity may be attributed primarily to the progressive removal of carbonaceous matter during carbonization and steam activation, resulting in a higher relative proportion of inorganic constituents in the remaining solid material. This trend does not necessarily indicate the formation of additional mineral matter but rather its relative enrichment as the organic fraction is progressively decomposed and gasified.

Additional elemental characterization by energy-dispersive X-ray spectroscopy (EDS) provided complementary information on the inorganic constituents present in selected activated carbons. In the sugar beet pulp-derived activated carbon (SBP) obtained under the 600/850 °C treatment sequence, Mg, Si, K, and Ca were detected, whereas the corresponding corn cob-derived material (CCM) exhibited a pronounced K signal, together with detectable Na, Mg, Al, and Si. Elemental mapping further revealed differences in the spatial distribution of these constituents, including locally enhanced Ca signals in SBP and K-rich regions in CCM. These observations indicate that the investigated biomass precursors retained distinct inorganic elemental signatures after thermal treatment.

Nevertheless, the EDS results represent localized, surface-sensitive elemental information and should not be interpreted as quantitative evidence of the overall mineral composition or as direct confirmation of pore blockage by inorganic constituents. The relationship between ash content and adsorption-related properties is therefore likely to depend on several interacting factors, including precursor composition, carbon consumption, and changes in the carbonaceous structure. The detailed SEM-EDS results and their relationship with BET specific surface area are discussed in Section 3.6.

Bulk density increased across the available activation sequences for every formulation. SHP had the highest values, while STM remained the least dense. This trend differs from the predominantly decreasing bulk density observed during carbonization but does not by itself demonstrate structural densification at the pore scale. The combined results distinguish several material profiles. SBP offered the highest measured BET surface area, CCM showed a strong increase in BET surface area with treatment severity, and APM combined comparatively low ash content with a near-neutral extract pH. STM showed limited adsorption performance after activation and a restricted usable temperature range under the tested conditions. These observations support formulation-specific selection rather than a single preferred thermal sequence for all feedstocks.

### 3.5. Trade-Off Between Adsorption Performance and Material Yield

The results demonstrate that increasing the severity of the thermal treatment improved the adsorption-related properties of most activated products, but this improvement was accompanied by a reduction in material recovery. This relationship reflects the fundamental mechanism of physical steam activation. At elevated temperatures, steam reacts with the carbon matrix according to the water–gas reaction, resulting in the removal of carbon atoms, the opening of blocked pores, and the development of new microporous and mesoporous structures. At the same time, progressive gasification inevitably decreases the mass of the solid product. Similar relationships between activation severity, pore development, and carbon yield have been reported for biomass-derived activated carbons obtained using steam or carbon dioxide [21,22,23].

The estimated overall yields, calculated from the product of the carbonization and activation yields, decreased with increasing process temperature. This tendency is consistent with the thermal decomposition of organic constituents during carbonization and the subsequent partial gasification of the carbonized matrix during steam activation. A higher carbonization temperature promotes the removal of volatile components and increases the fixed-carbon fraction but reduces the quantity of solid material remaining after the process. During activation, the effect is intensified because the activating agent reacts directly with the carbon skeleton. Similar decreases in solid yield with increasing carbonization temperature have been reported for lignocellulosic biomass, together with an increase in carbonization degree and structural stability [24,25].

The relationship between BET surface area and estimated overall yield differed considerably among the formulations investigated. Sugar beet pulp produced the highest BET values, reaching 459 m^2^/g in the 600/850 °C sequence, but the corresponding estimated overall yield decreased to 5.92%. The increase in BET surface area between the 500/700 and 600/850 °C sequences was only 1.5%, whereas the process was accompanied by a decrease in estimated yield and an increase in ash content. This result suggests that the highest temperature did not provide a proportional improvement in the adsorption potential of this precursor. Comparable diminishing effects at high activation severity have been described in studies of steam-activated biomass, where further burn-off can lead to pore widening, coalescence, or partial destruction of the most developed microporous domains [17,18].

The response of corn cob-based material was different. Increasing the treatment severity from 500/700 to 600/850 °C increased the BET surface area from 369 to 449 m^2^/g, while the estimated overall yield decreased from 7.74 to 7.31%. The relatively large increase in surface area at a comparatively moderate loss of yield indicates that corn cobs with molasses were highly responsive to steam activation under the investigated conditions. This behavior may be associated with the low initial ash content of the corn cob precursor and the high content of volatile matter, which favoured the formation of a reactive carbon matrix during carbonization. The suitability of corn cob and other agricultural residues as precursors for porous carbon materials has also been reported in studies focused on adsorption and environmental applications [26,27].

Sunflower husk pellets also showed a substantial increase in BET surface area, from 276 m^2^/g in the 400/600 °C sequence to 421 m^2^/g in the 600/850 °C sequence. However, the increase was accompanied by a gradual reduction in the estimated overall yield and an increase in ash content. The behavior of this material confirms that the development of porosity was enhanced by more intensive activation, although the process progressively concentrated the inorganic fraction in the remaining solid. Previous studies have demonstrated the suitability of sunflower-derived biomass for the preparation of activated carbons and have highlighted the influence of feedstock characteristics and thermal processing conditions on the properties of the resulting carbonaceous materials [28,29].

Apple pomace with molasses exhibited a more moderate response to increasing treatment severity. Its BET surface area increased from 375 to 431 m^2^/g, while the estimated overall yield decreased from 7.07 to 6.27%. The relatively high BET value obtained at the lowest activation sequence indicates that this precursor developed a substantial accessible surface area without the need for the most severe treatment. In addition, apple pomace produced activated materials with comparatively low ash content and an aqueous extract pH close to neutrality. This combination may be advantageous in applications where both adsorption capacity and limited mineral alkalinity are required. Previous studies have confirmed that apple pomace can be converted into functional carbonaceous adsorbents, although their physicochemical properties and potential applications depend on the applied preparation and modification procedures [30].

The straw-based formulation presented the least favourable balance between adsorption performance and material recovery. Its BET surface area increased only from 176 to 196 m^2^/g between the 400/600 and 500/700 °C sequences, while the estimated overall yield decreased from 5.25 to 4.61%. In the 600/850 °C sequence, the material underwent almost complete ashing and could not be characterized. This behaviour was associated with the relatively high ash content of the straw-derived products, which increased from 28.72 to 33.42% during activation. A high mineral fraction can reduce the proportion of reactive carbon available for pore development and can promote structural instability during severe thermal treatment. Similar limitations have been reported for biomass feedstocks with high ash contents, particularly agricultural residues containing substantial amounts of alkali and alkaline-earth minerals [24,31].

The comparison of the materials investigated indicates that the highest BET surface area should not be used as the sole criterion for selecting the optimal process conditions. A suitable activated carbon should combine an adequately developed pore structure with acceptable material recovery, moderate ash content, and sufficient structural stability. From this perspective, the 600/850 °C sequence was advantageous for corn cob-based material because it produced a substantial increase in BET surface area while maintaining a relatively high estimated overall yield. For sugar beet pulp, the 500/700 °C sequence may represent a more balanced condition, because the additional increase in BET obtained at 600/850 °C was small in relation to the associated loss of yield and increase in ash content. Apple pomace also showed favourable properties at intermediate or lower activation severity, whereas straw was unsuitable for the highest investigated temperature.

The estimated overall yield used in this analysis should be interpreted as a comparative process indicator rather than an absolute industrial yield. It was calculated from the reported mean yields of the carbonization and activation stages and assumes that both stages were referenced to consistent mass bases. For formulations containing molasses, the calculation also reflects the presence of the binder in the initial granulated feed. In addition, the absence of replicate-level yield data prevents statistical evaluation of the uncertainty associated with these estimates. Nevertheless, the comparison provides a useful basis for evaluating the balance between the development of adsorption properties and the preservation of the solid product.

The contrasting BET–yield relationships observed for SBP and CCM provided the basis for selecting these two materials for additional SEM-EDS characterization. The results presented in Section 3.6 offer complementary insight into their surface morphology and inorganic elemental composition, without implying a direct quantitative relationship between SEM-visible features and micropore development.

**Figure 10:**
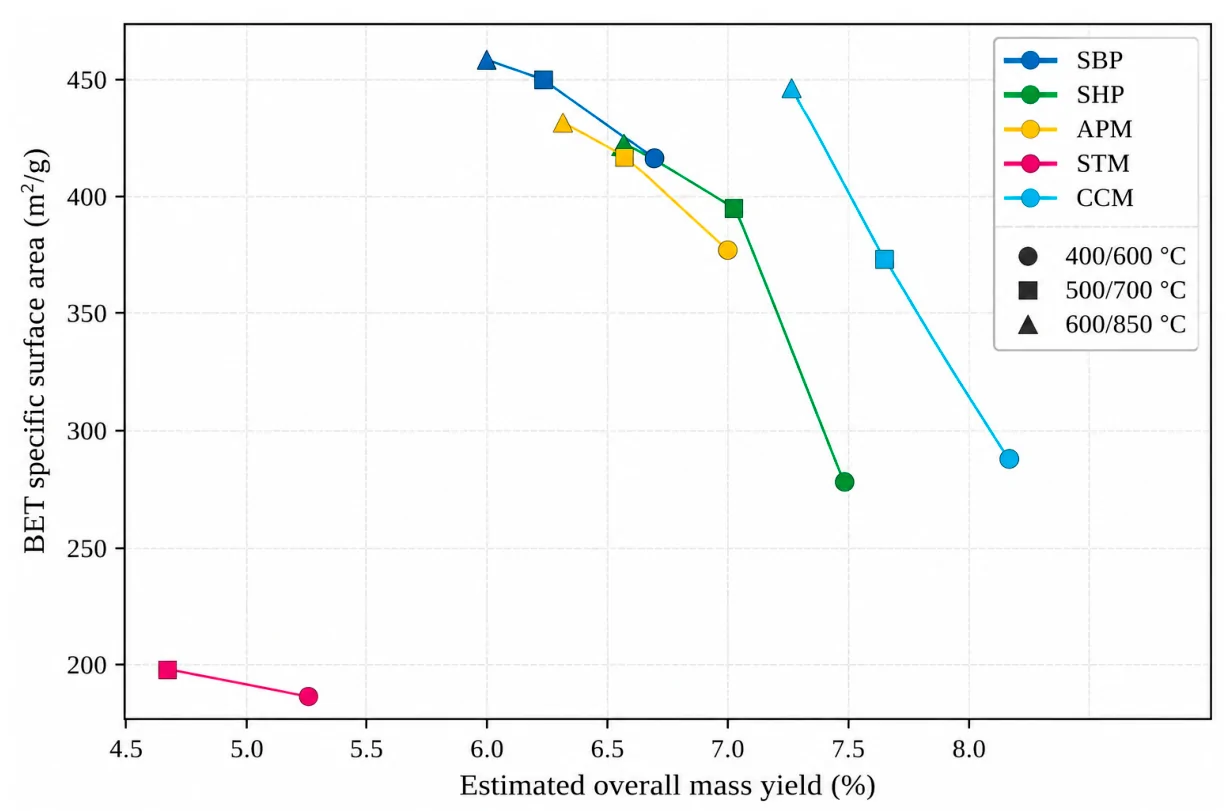
Relationship between BET specific surface area and estimated overall mass yield of activated carbons obtained under different carbonization/steam activation temperature sequences. Colours represent the biomass precursors, while marker shapes indicate the treatment sequences. Connecting lines link experimental treatments within each precursor formulation. The STM sample treated at 600/850 °C was excluded because extensive loss of the carbonaceous fraction prevented its physicochemical characterization.

Overall, Figure 10 confirms that the best material is not necessarily the material with the largest surface area. The scientifically and technologically relevant optimum lies on a compromise frontier, where the incremental gain in adsorption-related porosity remains commensurate with the additional loss of solid yield. In the present system, CCM at 600/850 °C offers the most favourable high-performance compromise, whereas SBP at 500/700 °C is preferable when minimising burn-off and preserving material yield are prioritised. These conclusions provide a rational basis for selecting operating conditions in subsequent adsorption tests and for comparing the environmental and process performance of the investigated agricultural and agri-food residues.

### 3.6. Morphological and Elemental Characterization of Selected Activated Carbons

**Figure 11:**
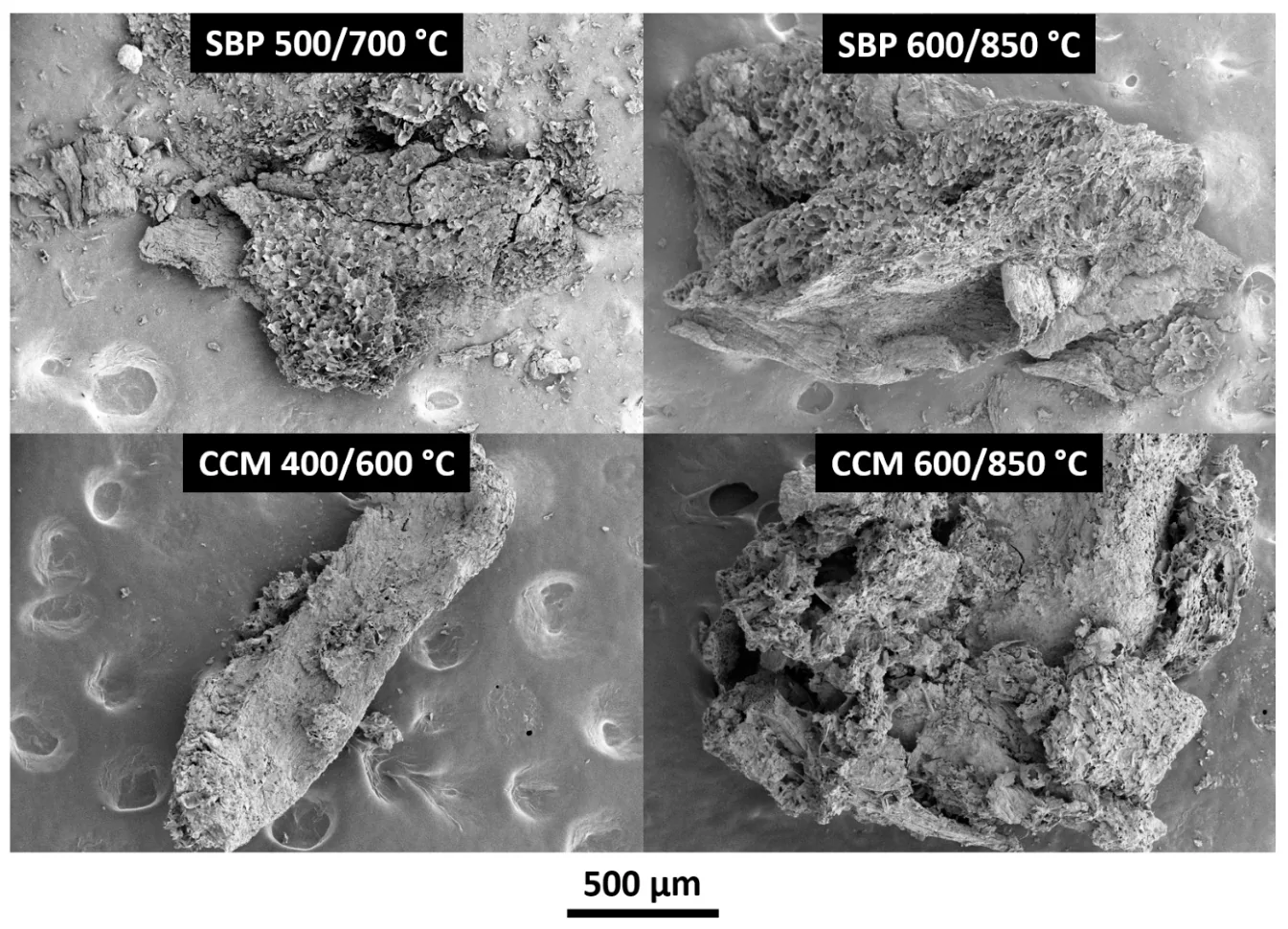
SEM micrographs of activated carbons derived from sugar beet pulp (SBP) and corn cobs with molasses (CCM) under different carbonization/steam activation temperature sequences.

**Figure 12:**
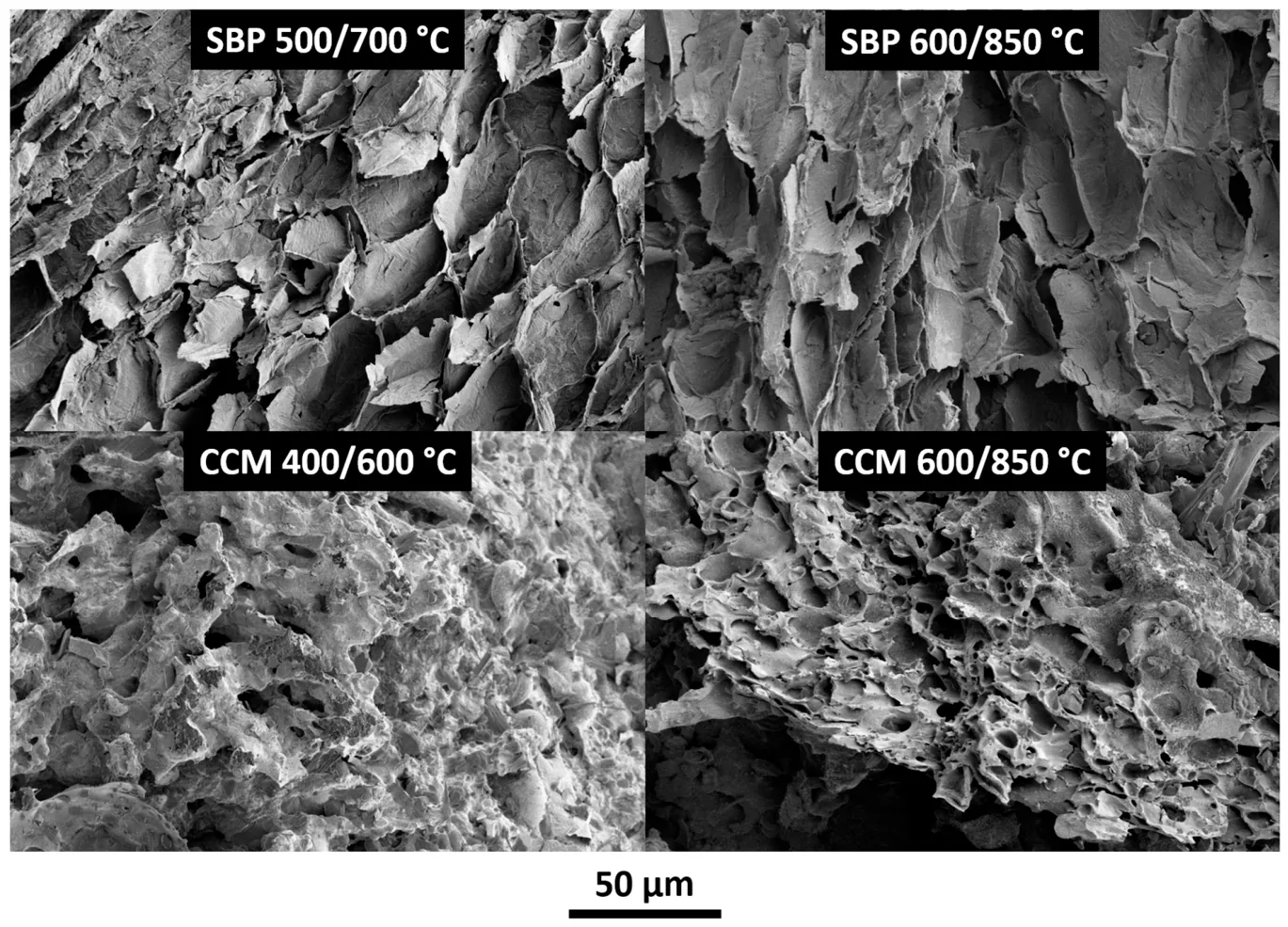
Higher-magnification SEM micrographs of selected activated carbons.

**Figure 13:**
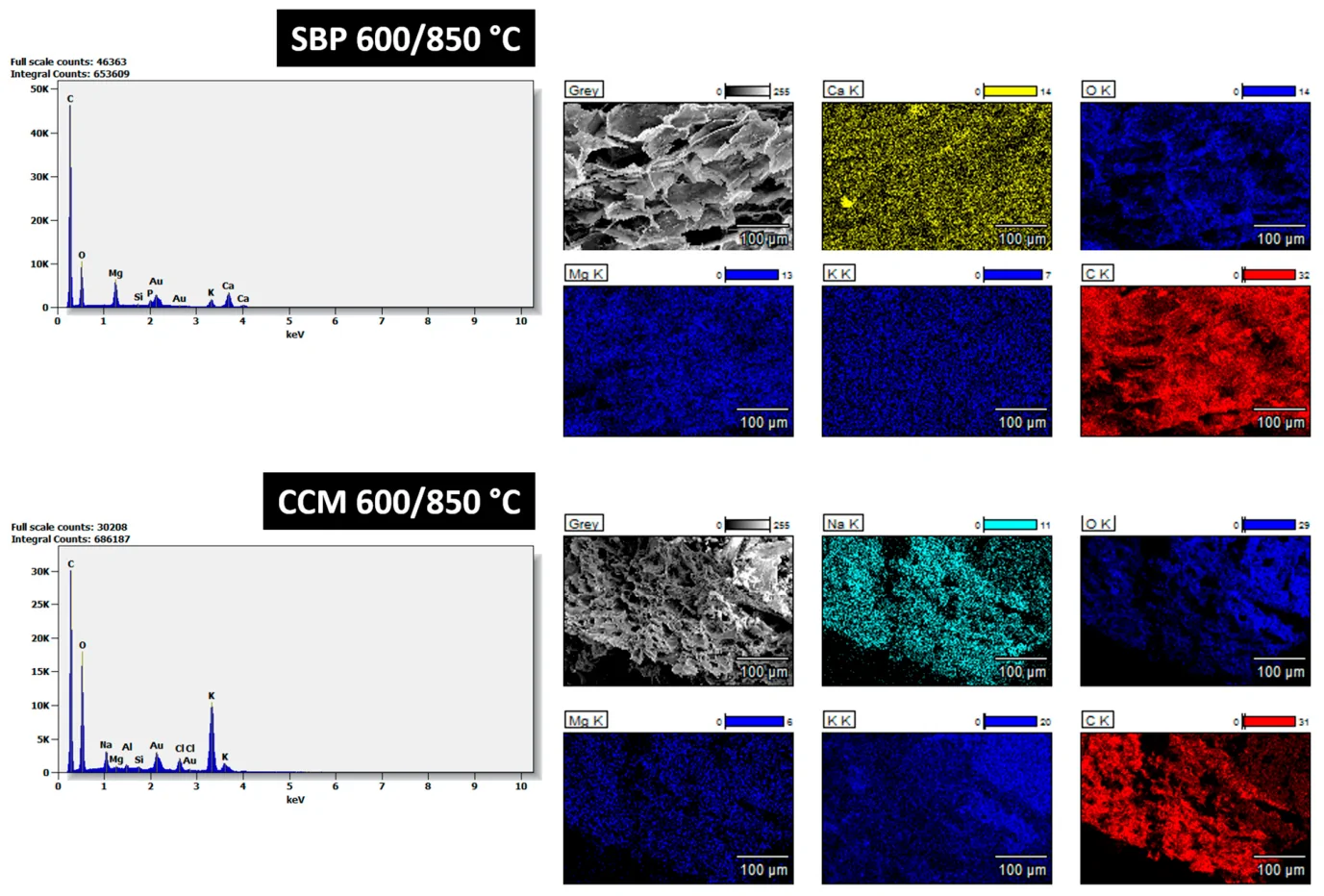
SEM-EDS characterization of activated carbons obtained from sugar beet pulp (SBP) and corn cobs with molasses (CCM), both subjected to carbonization at 600 °C followed by steam activation at 850 °C. The figure presents EDS spectra, corresponding SEM images, and elemental distribution maps of selected elements. Au signals originate from the conductive coating applied during SEM specimen preparation.

To provide additional information on the morphology and elemental composition of selected samples, scanning electron microscopy (SEM) and energy-dispersive X-ray spectroscopy (EDS) were used for the activated carbons derived from sugar beet pulp (SBP) and corn cobs with molasses (CCM). The selection was based on the two different responses to increasing carbonization and steam activation temperatures, as identified from BET specific surface area measurements (Figure 9). SEM micrographs obtained at different magnifications are presented in Figure 11 and Figure 12, while the corresponding EDS spectra and elemental distribution maps are shown in Figure 13.

In SEM observations, heterogeneous surface morphologies were revealed in both groups of analyzed activated carbons. Irregularly shaped particles, local fractures, and partially preserved structural features originating from the biomass precursors were identified. In the case of SBP, both the 500/700 °C and 600/850 °C samples exhibited a complex morphology, where cavities and fragmented carbonaceous structures were visible (Figure 11). Higher-magnification images (Figure 12) revealed numerous multilayered and fractured fragments of the structure. Similar morphological features in activated carbons obtained from sugar beet pulp were reported by Selinger et al. [32]. Nevertheless, although local differences in surface morphology were observed, no substantial or systematic changes could be identified between the two temperature sequences.

The described observations are consistent with findings from BET analysis, where similar specific surface area was found in the two discussed SBP samples (Figure 9). As discussed in earlier sections, this limited improvement was accompanied by a reduction in the estimated overall process yield. Similar effects were described by Zhang et al. [23], who showed that the increase in severity of the steam activation process may promote both formation and widening of the pores, without producing a further increase in specific surface area.

However, more severe morphological differences were seen in CCM-derived activated carbons. At 400/600 °C, the carbonaceous particles exhibited relatively compact, elongated fragments with rough surfaces and irregular openings (Figure 11 and Figure 12). Conversely, the sample obtained at 600/850 °C presented a more fragmented and open morphology, with numerous visible cavities and irregular voids (Figure 11 and Figure 12). This visual comparison corresponds to the increase in BET specific surface area from 286 to 449 m^2^/g (approximately 57%). Nevertheless, it should be emphasized that SEM provides information on particle morphology and larger features, while BET is strongly influenced by much smaller pores which cannot directly be pointed out in the presented micrographs.

Additionally, SEM analyses were performed on the SBP and CCM samples obtained at 600/850 °C. According to the presented results, differences in the inorganic elements were detected between the two analyzed materials (Figure 13). In both cases, carbon and oxygen produced the dominant signals. However, the SBP spectrum showed the presence of Mg, Si, K, and Ca. In contrast, the CCM spectrum exhibited a strong K signal, together with detectable amounts of Na, Mg, Al, and Si. Therefore, it can be stated that while being subjected to the same temperature sequence, activated carbons produced from different precursors retained distinct inorganic elemental signatures.

The elemental distribution maps provided further information on the uniformity of distribution of the detected elements. In the SBP sample, Ca was detected across much of the investigated particle area. Nevertheless, locally, its increased signal intensity was seen. On the other hand, K, which was uniformly distributed in SBP, in CCM tends to form larger, isolated K-rich areas, accompanied by an increased intensity in oxygen as well.

Overall, the SEM observations complemented physicochemical characteristics of the studied activated carbons, revealing a link between the morphology of the particles and trends seen in BET specific surface area measurements. The EDS results additionally demonstrated differences in the detected inorganic elements and their spatial distribution, particularly the noticeable Ca signal in SBP and the pronounced K signal in CCM. Together, these findings further emphasize the influence of biomass precursor selection on the properties of the resulting activated carbons.

### 3.7. Comparison with Commercial Activated Carbons and Practical Limitations

To provide a broader perspective on the practical potential of the prepared activated carbons, their adsorption-related physicochemical characteristics were compared with those of selected commercially available activated carbon products used in water treatment applications. Two commercial granular activated carbons, Jacobi AquaSorb 1000 and Chemviron FILTRASORB 400, were selected as reference materials because their technical datasheets provide information on both BET specific surface area and iodine number [28,29]. The comparison was based on manufacturer-reported typical properties rather than on additional experimental measurements performed in the present study. Among the investigated biomass-derived materials, sugar beet pulp-derived activated carbon (SBP) and corn cob-derived activated carbon prepared with molasses (CCM), both produced under the 600/850 °C carbonization/steam activation sequence, were selected for comparison because they exhibited the highest BET specific surface areas and iodine numbers. The corresponding results are summarized in Table 9.

The comparison revealed that both biomass-derived activated carbons exhibited lower BET specific surface areas and iodine numbers than the selected commercial reference products. The BET specific surface area of SBP (459 m^2^/g) corresponded to approximately 51% of that reported for AquaSorb 1000 and 44% of that reported for FILTRASORB 400. Similarly, CCM reached approximately 50% and 43% of the respective commercial BET values. A comparable relationship was observed for iodine adsorption. The iodine numbers of SBP and CCM corresponded to approximately 55% and 54%, respectively, of the value reported for AquaSorb 1000, and approximately 45% of the value reported for FILTRASORB 400. These differences indicate that the pore development and iodine uptake achieved under the investigated thermal processing conditions remained below the values characteristic of the selected commercial activated carbons. Nevertheless, the results confirm that agricultural and agri-food waste formulations can serve as precursors for producing carbonaceous materials with measurable iodine adsorption capacity and developed specific surface areas. It should be emphasized that the commercial products represent industrially manufactured granular activated carbons, whereas the materials investigated in this study were produced under laboratory-scale conditions and subsequently ground for physicochemical characterization. Differences in precursor composition, activation conditions, processing scale, particle size, and analytical procedures may influence the reported values. Consequently, the comparison cannot be used to establish equivalent adsorption performance or predict pollutant removal efficiency under actual water treatment conditions.

Furthermore, BET specific surface area and iodine number provide information on selected adsorption-related characteristics but do not directly determine adsorption kinetics, equilibrium capacity, or selectivity toward individual contaminants. A comprehensive assessment of the practical applicability of the prepared activated carbons would therefore require comparative adsorption experiments using commercial reference materials under identical conditions. Although the investigated biomass-derived activated carbons did not reach the adsorption-related parameters reported for the selected commercial products, the results provide a basis for further optimization of thermal processing conditions and precursor preparation. Future studies should focus on improving pore development while maintaining acceptable material yields and evaluating adsorption performance toward selected pollutants under standardized experimental conditions.

The regeneration and reusability of activated carbon are important factors determining its practical applicability and long-term economic performance. Regeneration efficiency depends on several factors, including the properties of the adsorbed compounds, the strength of adsorbate–adsorbent interactions, the regeneration method, and changes in the structure and surface chemistry of the carbon during repeated adsorption–desorption cycles. In the present study, regeneration experiments were not performed because the experimental programme focused on activated carbon production and physicochemical characterization rather than on the removal of a selected pollutant. Consequently, no conclusions regarding regeneration efficiency, adsorption capacity retention, or long-term operational stability can be drawn from the available results. Future studies should evaluate the regeneration performance of selected activated carbons under pollutant-specific conditions, considering both adsorption efficiency and structural stability over successive cycles.

## 4. Conclusions

The results demonstrated that the physicochemical and adsorption-related properties of activated carbons derived from agricultural and agri-food biowaste depended strongly on the biomass precursor and the applied carbonization/steam activation temperature sequence. Increasing thermal treatment severity generally improved the iodine number and BET specific surface area, although the magnitude of these changes differed considerably among the investigated materials.

The highest BET specific surface area (459 m^2^/g) and iodine number (477 mg/g) were obtained for sugar beet pulp after the 600/850 °C treatment sequence. Corn cobs with molasses also exhibited favourable properties, reaching 449 m^2^/g and 467 mg/g, respectively. However, improved adsorption-related characteristics were generally accompanied by reduced material yields and increased ash contents. Straw showed limited suitability under the investigated conditions, particularly at the highest treatment severity, which resulted in extensive loss of the carbonaceous fraction.

SEM observations provided complementary information on the precursor-dependent morphological characteristics of selected activated carbons. Sugar beet pulp-derived samples exhibited no substantial systematic morphological changes between the 500/700 and 600/850 °C treatment sequences, consistent with the limited additional increase in BET specific surface area. In contrast, corn cob-derived carbons showed more pronounced surface fragmentation and visible cavities at higher treatment severity, corresponding to a substantial increase in BET specific surface area. EDS analysis further revealed distinct inorganic elemental signatures, particularly the presence of Ca in sugar beet pulp-derived carbon and a pronounced K signal in corn cob-derived carbon.

The findings indicate that increasing thermal treatment severity does not necessarily provide a proportional improvement in material properties and that BET specific surface area should not be considered independently of process yield, ash content, and precursor characteristics. Moreover, the SEM-EDS results provide complementary morphological and elemental information but do not independently establish micropore-size distributions or adsorption performance toward specific contaminants.

Comparison with selected commercially available activated carbons indicated that the biomass-derived materials exhibited lower BET specific surface areas and iodine numbers than established industrial products. The highest-performing laboratory samples reached approximately 43–51% of the BET specific surface areas and 44–55% of the iodine numbers reported for the commercial references. Although this comparison highlights the need for further optimization of the preparation process, it also demonstrates the potential of agricultural and agri-food waste as alternative precursors for activated carbon production. The commercial comparison was based on manufacturers’ technical data and should not be interpreted as evidence of equivalent adsorption efficiency. Further studies involving standardized pollutant-specific adsorption experiments and direct comparisons with commercial adsorbents are required.

Overall, the study highlights the importance of precursor-specific optimization of carbonization and steam activation conditions. Further investigations should include direct adsorption experiments with target contaminants, detailed pore-size distribution analysis, and regeneration studies to assess the practical suitability of the obtained materials for environmental applications.

## Figures and Tables

**Table 1:** Amount of beet molasses used for the preparation of biomass mixtures.

Raw Material	Biomass Mass (kg)	Molasses Volume (mL)	Molasses Dose (mL·kg^−1^)
Apple pomace	2.0	950	475
Straw	2.0	1500	750
Corn cobs	2.0	1200	600

**Table 2:** Carbonization and steam activation conditions used to produce carbonaceous materials.

Variant	Carbonization Temperature (°C)	Carbonization Time (min)	Activation Temperature (°C)	Activation Time (min)
I	400	60	600	60
II	500	60	700	60
III	600	60	850	60

**Table 3:** Mean yield of the biomass waste pyrolysis process (%).

Carbonization Temperature (°C)	Sugar Beet Pulp	Sunflower Husk Pellets	Apple Pomace	Ground Straw	Ground Corn Cobs
400	19.6	22.3	23.1	18.6	24.1
500	18.7	21.6	22.4	17.4	23.1
600	18.1	20.5	21.7	16.5	22.3

**Table 4:** Mean yield of the biomass waste activation process (%).

Activation Temperature (°C)	Sugar Beet Pulp	Sunflower Husk Pellets	Apple Pomace	Ground Straw	Ground Corn Cobs
600	34.0	33.6	30.6	28.2	34.2
700	33.1	32.8	29.4	26.5	33.5
850	32.7	31.9	28.9	—	32.8

**Table 5:** Moisture, ash, and volatile matter contents of the biomass feedstocks prior to carbonization. (mean ± SD, *n* = 3).

Feedstock	Moisture Content (%)	Ash Content (%)	Volatile Matter Content (%)
Sugar beet pulp	6.37 ± 0.199	3.01 ± 0.098	80.97 ± 3.150
Sunflower husk pellets	8.16 ± 0.162	1.96 ± 0.033	79.67 ± 2.836
Apple pomace	5.83 ± 0.136	1.81 ± 0.044	77.80 ± 2.754
Ground straw	7.29 ± 0.143	3.67 ± 0.138	80.52 ± 2.198
Ground corn cobs	7.91 ± 0.133	0.66 ± 0.025	81.82 ± 2.397

**Table 6:** Physicochemical characteristics of sugar beet molasses used as a binder.

Parameter	Unit	Value
Moisture content	%	22.1
Refractometric soluble solids content	Brix	79.9
pH	—	6.2
Dynamic viscosity	mPa·s	446

Note: The Brix value is a refractometric indicator of soluble solids and does not represent a selective determination of sugar content. The temperature corresponding to the reported viscosity value was not specified in the source results. Dynamic viscosity expressed in mPa·s is numerically equivalent to cP.

**Table 7:** Complementary physicochemical properties of the chars obtained at different carbonization temperatures (mean ± SD, *n* = 3).

Formulation	Carbonization Temperature (°C)	Ash (%)	Volatile Matter (%)	Bulk Density (g/L)
SBP	400	8.81 ± 0.22	32.82 ± 0.98	220 ± 5.28
SBP	500	10.33 ± 0.31	20.62 ± 0.45	208 ± 7.28
SBP	600	11.17 ± 0.20	17.88 ± 0.63	196 ± 4.90
SHP	400	7.89 ± 0.28	21.55 ± 0.39	274 ± 8.22
SHP	500	8.53 ± 0.19	13.84 ± 0.53	255 ± 5.87
SHP	600	8.67 ± 0.26	10.81 ± 0.24	231 ± 8.09
APM	400	7.22 ± 0.17	27.41 ± 0.82	202 ± 4.04
APM	500	7.71 ± 0.29	20.35 ± 0.41	205 ± 6.15
APM	600	8.89 ± 0.22	17.68 ± 0.62	206 ± 3.71
STM	400	15.25 ± 0.53	25.69 ± 0.64	119 ± 2.38
STM	500	16.65 ± 0.42	17.76 ± 0.53	109 ± 3.82
STM	600	20.25 ± 0.61	12.33 ± 0.25	108 ± 2.70
CCM	400	8.64 ± 0.17	23.76 ± 0.71	192 ± 5.76
CCM	500	10.34 ± 0.36	12.63 ± 0.25	187 ± 4.11
CCM	600	11.94 ± 0.30	10.81 ± 0.38	183 ± 6.41

SBP: sugar beet pulp; SHP: sunflower husk pellets; APM: apple pomace with molasses; STM: straw with molasses; CCM: corn cobs with molasses.

**Table 8:** Physicochemical properties of activated carbons obtained under paired carbonization/steam activation temperature sequences. Results are expressed as mean ± SD (*n* = 3) for ash content, volatile matter content, moisture content, and bulk density. The pH values are presented as arithmetic means of duplicate determinations.

Formulation	Temperature Pair (°C)	Ash (%)	Volatile Matter (%)	Moisture (%)	Bulk Density (g/L)	Extract pH
SBP	400/600	12.77 ± 0.51	11.45 ± 0.41	2.41 ± 0.10	204 ± 8.16	8.48
SBP	500/700	14.55 ± 0.22	11.15 ± 0.22	2.35 ± 0.07	215 ± 3.23	8.52
SBP	600/850	16.52 ± 0.25	10.31 ± 0.35	2.32 ± 0.03	221 ± 6.84	8.54
SHP	400/600	10.51 ± 0.42	10.92 ± 0.19	3.01 ± 0.12	240 ± 3.60	8.54
SHP	500/700	14.36 ± 0.22	9.62 ± 0.14	2.68 ± 0.19	245 ± 9.81	8.66
SHP	600/850	14.86 ± 0.59	8.39 ± 0.13	2.44 ± 0.04	251 ± 10.04	9.27
APM	400/600	10.94 ± 0.16	14.58 ± 0.31	2.11 ± 0.08	202 ± 8.08	7.21
APM	500/700	11.55 ± 0.30	14.31 ± 0.26	2.06 ± 0.06	217 ± 5.62	7.26
APM	600/850	11.78 ± 0.18	14.31 ± 0.57	2.03 ± 0.08	232 ± 9.28	7.31
STM	400/600	28.72 ± 1.15	13.84 ± 0.21	2.01 ± 0.09	119 ± 2.79	8.41
STM	500/700	33.42 ± 1.34	13.96 ± 0.56	1.99 ± 0.11	124 ± 4.96	8.41
STM	600/850	ND	ND	ND	ND	ND
CCM	400/600	18.12 ± 0.72	14.73 ± 0.22	2.18 ± 0.03	186 ± 2.79	8.40
CCM	500/700	18.33 ± 0.27	14.27 ± 0.57	2.08 ± 0.08	191 ± 4.61	8.68
CCM	600/850	19.60 ± 0.78	13.41 ± 0.20	2.01 ± 0.09	221 ± 10.12	9.00

SBP: sugar beet pulp; SHP: sunflower husk pellets; APM: apple pomace with molasses; STM: straw with molasses; CCM: corn cobs with molasses. ND: not determined because of extensive loss of the carbonaceous fraction.

**Table 9:** Comparison of the adsorption-related physicochemical characteristics of selected biomass-derived activated carbons with commercial activated carbon products.

Activated Carbon	Raw Material	BET Specific Surface Area (m^2^/g)	Iodine Number (mg/g)	Reference
SBP (600/850 °C)	Sugar beet pulp	459	477	This study
CCM (600/850 °C)	Corn cobs with molasses	449	467	This study
Jacobi AquaSorb 1000	Coal-based activated carbon	900	870	[33]
Chemviron FILTRASORB 400	Coal-based activated carbon	1050	1050	[34]

**Note:** SBP, sugar beet pulp-derived activated carbon; CCM, corn cob-derived activated carbon prepared with molasses. Temperatures refer to carbonization/steam activation. Commercial values are typical properties reported in the manufacturers’ technical datasheets [33,34], not results measured in this study. This indicative comparison concerns BET specific surface area and iodine number; it does not establish equivalent adsorption efficiency for specific pollutants.

## Data Availability

The experimental data supporting the findings of this study were generated by the authors and are presented in the article. Additional information regarding the experimental results may be obtained from the corresponding author upon reasonable request. The reference data for commercial activated carbons were obtained from the manufacturers’ technical datasheets cited in the manuscript.
